# Sweet potato (*Ipomoea batatas* L.) genotype selection using advanced indices and statistical models: A multi-year approach

**DOI:** 10.1016/j.heliyon.2024.e31569

**Published:** 2024-05-20

**Authors:** Zakaria Alam, Sanjida Akter, Md Anwar Hossain Khan, Md Iqbal Hossain, Md Nurul Amin, Avijit Biswas, Ebna Habib Md Shofiur Rahaman, Mir Aszad Ali, Debashish Chanda, Md Hasan Sofiur Rahman, Md Abu Kawochar, Md Shamshul Alam, Mohammad Mainuddin Molla, Md Monirul Islam, M.A.H.S. Jahan, Md Zulfikar Haider Prodhan, Md Monjurul Kadir, Debasish Sarker

**Affiliations:** a Bangladesh Agricultural Research Institute (BARI), Gazipur, 1701, Bangladesh; b Bangladesh Rice Research Institute (BRRI), Gazipur, 1701, Bangladesh; c International Potato Centre (CIP), Bangladesh Office, Dhaka, 1230, Bangladesh; d Bangladesh Institute of Nuclear Agriculture (BINA), Mymensingh, 2202, Bangladesh

**Keywords:** Sweet potato selection, Stability, MGIDI, FAI-BLUP, WAAS, MTSI

## Abstract

In Bangladesh, sweet potato holds the fourth position as a crucial carbohydrate source, trailing rice, wheat, and potato. However, locally grown sweet potato varieties often display limited stability and yield. To tackle this challenge, diverse selection methods and statistical models were utilized to pinpoint sweet potato genotypes showcasing both stability and superior yield and quality traits. In the initial two years, multiple selection methods were employed to narrow down the collections based on preferences for yield and its contributing traits. Subsequently, a multi-environment trial (MET) was conducted in the following year to pinpoint superior and stable genotypes with desirable yield and quality characteristics. An integrated approach involving the Multi-Trait Genotype Ideotype Distance Index (MGIDI), Factor Analysis and Ideotype-Design (FAI-BLUP), and Smith-Hazel Index (SH) led to the identification of 71 superior sweet potato genotypes out of a total of 351 in the initial growing season. In the subsequent season, the MGIDI selection index was applied to the 71 genotypes, resulting in the selection of 11 top-performing genotypes. This selection process was complemented by a detailed analysis of the strengths and weaknesses of the selected genotypes. In the MET, the mixed effect model, specifically the linear mixed model (LMM), identified significant genotypic and genotype-environment interaction (GEI) variances. This points to elevated heritability and selection accuracy, ultimately boosting the model's reliability. By combining the strengths of LMM and additive main effects and multiplicative interaction (AMMI), the best linear unbiased prediction (BLUP) index identified H20 as the top-performing genotype for marketable root yield (MRY), H37 for dry weight of root (DW), H8 for beta carotene (BC) and H41 for vitamin c (VC). These genotypes surpassed the overall average in the WAAS index. For simultaneous stability and high performance, the WAASBY index selected H37 for MRY, H6 for DW, H61 for BC, and H3 for VC. Finally, genotypes H3 and H20 were selected using multi-trait stability index (MTSI), as they possessed high performance and stability. Based on the selection sense, the objective has been achieved with regards to the trait MRW, which serves as a major criterion for a superior variety of sweet potato.

## 1 Introduction

Globally, the cultivation of sweet potato (*Ipomoea batatas* L.) has grown over time, and the most recent CIP (International Potato Centre) figures indicate that 150 million tons of sweet potatoes are produced globally each year. China (more than 77.38 million tons) and other Asian nations including Indonesia, Japan, and Korea account for the majority of global production [1]. In Bangladesh, the sweet potato production also showed a progressive trend over the last decade. Between 2018-19 and 2020-21, a noteworthy upsurge of 16 % was observed in the aggregate yield of sweet potatoes. This yield growth can be attributed to the introduction and adoption of improved sweet potato varieties [2]. Improved sweet potato varieties are rich in vitamin A, with a maximum of ten times the amount found in local sweet potatoes [3] and potential yield above 40 tons per hectare [1,[3], [4], [5]]. Nevertheless, the locally cultivated sweet potatoes of Bangladesh exhibit an average yield of approximately 10.50 tons per hectare and demonstrate poor stability [6]. On the other hand, the average yield of sweet potato in America, China and Portugal is 21.65 tons per hectare [7]. Therefore, sweet potato breeders are pursuing genetic improvement to identify new alleles and traits that contribute to yield and quality, with the aim of incorporating them into the genetic background. On the other hand, GEI hold immense significance and an utmost importance in the realm of sweet potato breeding for multi environment trials (METs).

The utilization of mixed effect models, specially the linear mixed model (LMM) is often employed in the selection processes for determining the predicted genotypic values of important breeding objective traits and enhancing the efficiency of selection [[8], [9], [10], [11]]. To achieve this objective, the restricted maximum likelihood (REML) and best linear unbiased prediction (BLUP) have been utilized as effective selection models for estimating variance components and predicting genotypic values, respectively [12]. Several effective selection indices, such as the multi-trait genotype ideotype index (MGIDI) [13], factor analysis and ideotype-design (FAI-BLUP) index [14], and Smith-Hazel (SH) index [15,16], have been used in breeding programs of crops [12,17,18]. Pour-Aboughadareh & Poczai [19] has recently introduced a cutting-edge tool that enables breeders to efficiently select the most desirable genotypes by considering the interplay between MGIDI, FAI-BLUP, and SH selection indices.

Several models have been developed to analyze the data obtained from METs. Among these models, the additive main effects and multiplicative interaction (AMMI) model holds significant importance. In addition, a novel index named weighted average absolute scores (WAAS) [20], which is based on the AMMI model, has been incorporated into the indices for METs analysis. Beside AMMI, the GGE biplot graphical model has also been widely utilized to identify environments and winning genotypes within specific environments [21,22]. Additionally, the GGE biplot method has been employed to identify stable genotypes and determine their interaction with yield and environments. Furthermore, the best likelihood unbiased prediction (BLUP) method is proficient in estimating the genotypes’ mean yield in mixed models for METs [23,24]. To enhance the analysis, a combined index named as WAASB (weighted average absolute scores of BLUPs) has been developed by integrating WAAS model and BLUP [20]. Plant breeders, in their pursuit to identify and introduce new crop varieties while reducing the impact of GEI, consider both yield and stability simultaneously. Therefore, an index named WAASBY has been introduced, which combines WAASB and yield (Y) [20]. Olivoto et al. [20] provides an exposition on the theoretical underpinnings of the multi-trait stability index (MTSI), taking into account the combined influence of both effects (fixed and random). This index enables the superior genotypes selection in METs based on high yield and stability, taking into account multiple traits.

Hence, the primary goal is to develop and enhance superior sweet potato varieties with broader adaptability to thrive in diverse environmental conditions. In this study, 351 sweet potato genotypes were initially cultivated in the first year, aiming to identify superior genotypes based on yield and related traits using various advanced selection indices in an augmented trial. Subsequently, 71 superior genotypes out of the initial 351 were selected and grown in the second year using a randomized complete block design (RCBD) to streamline the materials with a 15 % selection pressure. In the third year, consideration was given to GEI by employing METs to observe their influence on the yield and quality of the sweet potato roots.

## 2 Materials and methods

### 2.1 Experimental materials, locations and design

A total of 351 sweet potato genotypes were procured from diverse geographical origins, encompassing Mozambique, Japan, Indonesia, and Bangladesh (Supplementary Table S1). Supplementary Table S2 presents a comprehensive overview of these collected genotypes, accompanied by detailed information regarding the climatic conditions of their respective origins.

The experiment was conducted in Bangladesh at the districts of Gazipur, Bogura and Jamalpur. The study site is situated between 23.6850° N and 90.3563° E latitude, at an elevation of 10 m above sea level in the Coastal South, and 105 m above sea level in the North. The monthly average temperature, rainfall, relative humidity and sunshine data of the research area are presented in Supplementary Table S3. The selection of the region was based on its representation of favorable sweet potato growth areas and suitable agroecology in Bangladesh. The physico-chemical properties of soil in three locations are presented in Supplementary Table S4 [1].

The cultivation of the collected genotypes spanned three consecutive growing seasons. During the initial season, the 351 genotypes were sown utilizing an augmented experimental design at the Bogura location. This design included four check varieties, namely BARI Mistialu-8, BARI Mistialu-12, BARI Mistialu-14, and BARI Mistialu-15. Ten vines of each genotype were planted in a single row, and the experimental field comprised ten blocks. Each block featured 35 non-replicated genotypes (except for one block with 36 genotypes), along with four replicated checks.

In the subsequent season at the same location, the selection process persisted with 71 selected genotypes, employing a randomized complete block design (RCBD) incorporating four check varieties (BARI Mistialu-8, BARI Mistialu-12, BARI Mistialu-14, and BARI Mistialu-15). Similar to the prior season, ten vines of each genotype were planted in a single row, and three rows of one genotype constituted a single plot. These established plots were then replicated three times.

During the final season, the RCBD was employed with 10 selected genotypes and one check variety (BARI Mistialu-12) across three locations: Gazipur, Bogura, and Jamalpur. The only deviation from the previous year's selection process was the inclusion of five rows per genotype within each plot, as opposed to three rows.

### 2.2 Management practices, harvesting and data collection

The management practices for sweet potato were done following a standard procedure of Alam et al. [1]. The harvesting took place after 130 days after vine planting, wherein each genotype consisted of 10 plants. In order to conduct a thorough quality analysis, the laboratory of Postharvest Technology Division at BARI collected and examined the storage roots of each genotype, each weighing 500 g. The following data was gathered in accordance with the methodology outlined by Alam et al. [1]: average foliage fresh weight per plant (FW) (g), average storage root number per plant (RN), average storage root weight per plant (RW) (g), marketable storage root number per plant (MRN), marketable storage root weight per plant (MRW) (g), average storage root length (RL) (cm), average storage root diameter (RD) (cm), marketable storage root yield (MRY) (t/ha), and dry weight of storage roots (DW) (%).

Modifications were made to methodology of Molla et al. [25] to quantify beta-carotene content (BC) (mg/100g). Freeze-dried sweet potato storage roots from Fisher Scientific Ltd. in the UK were mixed with acetone and petroleum ether. The resulting solution was then purified using metabolic potassium hydroxide (KOH), distilled water, and anhydrous sodium sulfate. The absorbance at 765 nm was measured using a UV–Vis Double Beam Spectrophotometer against petroleum ether as a blank.

The determination of ascorbic acid (vitamin C) content (VC) (mg/100g) was carried out following the procedure outlined by Ranganna [26]. This involved blending 10 g of samples for 2 min and homogenizing the mixture with 50 mL of 3 % cold *meta*-phosphoric (HPO_3_) acid. The homogenate was filtered to obtain clear supernatant samples for ascorbic acid assay. Aliquot samples were titrated with 2,6-dichlorophenolindophenol solution. The titer value was recorded for each sample. The 2,6-dichlorophenolindophenol solution was calibrated using an ascorbic acid standard solution. Results were expressed in mg/100g.

### 2.3 Statistical analysis

The analysis of variance (ANOVA) for yield and yield-contributing traits in the first two years was conducted using the augmentedRCBD and agricolae packages in R studio [27]. Mean values of sweet potato genotypes were separated through the least significant difference test (LSD). To facilitate genotype selection and assess stability, various indexing models were employed, utilizing multiple equations outlined in Table 1 and analyzed using the metan package in R studio [27].

**Table 1:** Selection and stability indices used in the present study.

No.	Index	Equation	Reference
**1**	**LMM**	y=Xb+Zu+e	[28]
**2**	**Ideotype design and rescaling the traits**	$rX_{ij}=\frac{\eta_{nj}-\varphi_{nj}}{\eta_{oj}-\varphi_{oj}}\times \left(\theta_{ij}-\eta_{0j}\right)+\eta_{nj}$	
**3**	**Broad-sense heritability**$\left(h^{2}\right)$	$h^{2}={\hat{\sigma }}_{\alpha }^{2}/\left({\hat{\sigma }}_{\alpha }^{2}+{\hat{\sigma }}_{\epsilon }^{2}/r\right)$	
**4**	**SG (%)**	$SG\left(\%\right)=\frac{\left({\bar{X}}_{s}-{\bar{X}}_{o}\right)\times h^{2}}{{\bar{X}}_{o}}\times 100$	
**5**	**MGIDI**	${MGIDI}_{i}={\left[\sum_{j=1}^{f}{\left(\gamma_{ij}-\gamma_{j}\right)}^{2}\right]}^{0.5}$	
**6**	**Strength and weaknesses of selected genotypes (MGIDI)**	$\omega_{ij}=\frac{\sqrt{D_{\mathrm{i}j}^{2}}}{\sum_{j=1}^{f}\sqrt{D_{ij}^{2}}}$	
**7**	**SH**	b = P^−1^Aw	[14]
**8**	**FAI-BLUP**	$P_{ij}=\frac{\frac{1}{d_{ij}}}{\sum_{i=1:j=1}^{i=n:j=m}\frac{1}{d_{ij}}}$	
**9**	**Factor analysis**	X=μ+Lf+ε	[28]
**10**	**Factor loadings**	$F=Z{\left(A^{T}R^{-1}\right)}^{T}$	
**11**	**AMMI**	Y_ij_ = μ+G_i_ + E_j_+ $\sum_{k=1}^{n}\lambda$_k_α_ik_γ_jk_+ε_ij_	[29]
**12**	**WAAS**	${\text{WAAS}}_{i}=\frac{\sum_{k=1}^{P}\;\left|{\text{IPCA}}_{\text{ik}}\times {\text{EP}}_{k}\right|}{\sum_{k=1}^{P}\;{\text{EP}}_{k}}$	
**13**	**WAASB**	${\text{WAASB}}_{i}=\frac{\sum_{k=1}^{P}\;\left|{\text{IPCA}}_{\text{ik}}\times {\text{EP}}_{k}\right|}{\sum_{k=1}^{P}\;{\text{EP}}_{k}}$	
**14**	**WAASBY**	$\begin{array}{rr} {\text{WAASBY}}_{i} & =\frac{\left(rG_{g}\times \theta_{Y}\right)+\left(rW_{g}\times \theta_{S}\right)}{\theta_{Y}+\theta_{S}} \end{array}$	
**15**	**GGE biplot**	$Y_{\text{ij}}-\mu -\beta_{j}=\lambda_{1}\xi_{i1}\eta_{j1}+\lambda_{2}\xi_{i2}\eta_{j2}+\varepsilon_{\text{ij}}$	
**16**	**MTSI**	${\text{MTSI}}_{i}={\left[\sum_{j=1}^{f}\;{\left(\gamma_{ij}-\gamma_{j}\right)}^{2}\right]}^{0.5}$	[20]

Before initiating selections in the first year, the significance of each trait concerning genotypes was determined through a Likelihood Ratio Test (LRT) at a probability level of p < 0.05, using a Linear Mixed Model (LMM) equation (Equation 1). This involved acquiring Best Linear Unbiased Predictors (BLUPs) of 355 genotypes for each trait to populate a two-way table for ideotype design and rescaling (Equation 2). The resulting data were used to calculate Venn plots for MGIDI (Equation 5), FAI-BLUP (Equation 7), and SH (Equation 8) indexes, aiming for a positive selection gain (SG) (Equation 4) for all traits with a 28 % selection intensity (SI), including the broad-sense heritability (h^2^) (Equation 3). In the second year, traits RN, RW, RL, and RD were targeted with a weighted average trait performance, and the objective was a positive SG for MGIDI with a 15 % SI of 75 genotypes. In the third year, the MTSI was calculated, considering various yield and quality traits with high performance and a positive selection goal for all traits (Equation 16) of 11 genotypes. Factor analysis and factorial loadings were computed to group studied traits, identifying strengths and weaknesses among selected genotypes (Equation 6). The AMMI (Equation 11), WAAS (Equation 12), WAASB (Equation 13), and WAASBY (Equation 14) stability models were analyzed and graphically presented using R studio. The GGE biplot graphical model was employed to identify environments and winning genotypes within specific environments (Equation 15). Within METs, genetic parameters such as genotypic variance, GEI variance, residual variance, heritability, GEI correlation, and selection accuracy were computed using a mixed effect model namely LMM. This model was applied within a randomized complete block design, where replicates were treated as fixed factors, and genotypes were regarded as random factors.

## 3 Result and discussions

### 3.1 First year selection trial

#### 3.1.1 Estimation of variations among genotypes

The statistical examination of variance for the five agronomic traits (FW, RN, RW, MRN and MRW) related to yield is demonstrated in Table 2. The extent of diversity, as indicated by the coefficient of variation, amongst the genotypes investigated in the present study (Table 2) ranged from 35.5 % to 49.2 %. This implies that the presence of exceptional cases (outliers) among the genotypes under examination was not particularly prevalent. The check varieties exhibited notable distinctions (p < 0.05) in terms of FW, RW and MRW (Table 2). Moreover, the genotypes, including and excluding the checks, exhibited a considerable impact (p < 0.05) on FW, RN, RW, MRN and MRW (Table 2). The dissimilarities and mean differences were observed among the genotypes under investigation provide valuable insights into their diversity (Supplementary Table S5). The observed variations between the genotypes analyzed will contribute to subsequent selections made from an extensive collection of genotypes. Additionally, breeders must bear in mind that the production and yield of sweet potatoes are influenced by a multitude of factors, encompassing variety, nutrient accessibility, resource absorption and utilization [30,31]. Various genotypes of sweet potatoes exhibit distinct nutrient requirements and patterns of resource uptake and utilization. These factors significantly impact the yield of their roots, ultimately playing a crucial role in determining both the quantity and quality of the crop [32,33].

**Table 2:** Analysis of variance of 351 sweet potato genotypes investigated with four check varieties in an augmented experimental design.

Source of variation	df	Mean sum of square (MSS)				
		FW	RN	RW	MRN	MRW
Genotype (unadjusted)	354	78605**	13.341**	110952**	2.596*	84155**
Block (adjusted)	9	67038**	4.4152^ns^	219264***	8.188***	204954***
Check	3	161885***	13.0252^ns^	156124*	2.294^ns^	150795*
Genotype (without check)	350	77893***	13.3142**	103796**	2.409*	78201**
Check + Genotype	1	78150^ns^	23.6363^ns^	2480134***	68.757***	1968213***
Residuals	36	19423	6.0614	50401	1.398	38331
CV (%)		35.5	38.9	41.8	46.3	49.2

^df^ degrees of freedom, ^CV^ coefficient of variation, ***significant at p < 0.001, **significant at p < 0.01, *significant at p < 0.05, ^NS^ non-significant, ^FW^ average foliage fresh weight per plant (g), ^RN^ average storage root number per plant, ^RW^ average storage root weight per plant (g), ^MRN^ marketable storage root number per plant and ^MRW^ marketable storage root weight per plant (g).

#### 3.1.2 Selection of high-performance genotypes

The factor analysis and selection gain comparison for MGIDI, SH and FAI_BLUP index is presented in Table 3. In all the indices, sense of selection increased and the goal was fulfilled for selection. The uniqueness was highest in FW measuring 0.54. The lowest uniqueness trait was RW (0.05). All the studied traits have high broad-sense heritability in all three selection indices with a considerable amount of total selection gain (115.94–120.70). The selection process takes into account through the disparities among characteristics in relation to their level of hereditary influence. Several research findings have demonstrated that the selection can utilize the existing genetic variations possessing a certain level of heritability in order to enhance the value of specific traits [34,35]. On the other hand, the interrelation between heritability and selection gain is a matter of concern. An illustration of this can be seen in our findings, where the broad sense heritability of FW demonstrated a high percentage, which in turn resulted in a higher value for selection gain (Table 3). Similar outcomes have been reported in multiple studies pertaining to various traits [[36], [37], [38]]. In order to comprehend the advantages of selection, evaluating the selection gain with heritability of traits could serve to enhance crop improvement programs.

**Table 3:** Factor analysis, broad-sense heritability (h^2^), uniqueness and selection gain (SG) calculation through MGIDI, SH and FAI-BLUP selection index of 71 high performing genotypes.

Traits	Factors	Sense	Goal	h^2^	Uniqueness	SG (%)		
						MGIDI	SH	FAI-BLUP
FW	1	Increase	100	0.96	0.54	33.10	49.39	33.10
RN	1	Increase	100	0.92	0.50	20.00	17.19	20.00
RW	1	Increase	100	0.87	0.05	22.90	18.06	22.90
MRN	1	Increase	100	0.83	0.18	17.30	10.00	17.30
MRW	1	Increase	100	0.87	0.14	27.40	21.30	27.40
Total	–	–	–	–	–	120.70	115.94	120.70

^MGIDI^ multi-trait genotype ideotype index, ^SH^ Smith-Hazel and ^FAI−BLUP^ factor analysis and ideotype-design index.

**Fig. 1:**
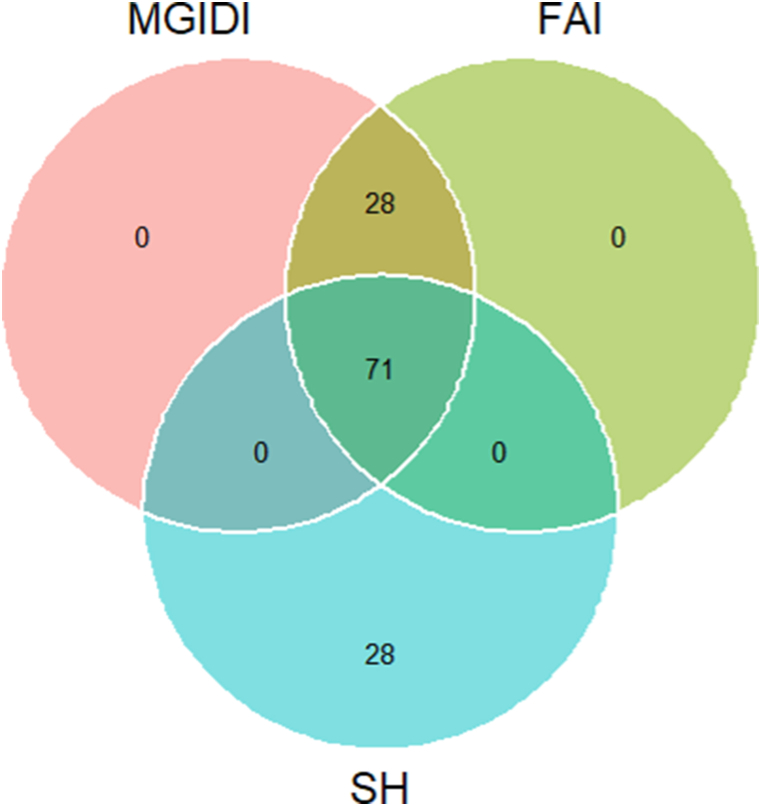
Selection of genotypes with a venn plot combination of MGIDI (multi-trait genotype ideotype index), SH (Smith-Hazel) and FAI-BLUP (factor analysis and ideotype-design) index.

In all three selection indices, a total of 99 genotypes were selected (Fig. 1). Among the selected genotypes, 71 genotypes were common in three indices (Fig. 1). To identify the superior genotypes, taking into account various characteristics, Olivoto and Nardino [28] utilized the MGIDI method, while Rocha et al. [14] utilized FAI-BLUP, and Smith [16] and Hazel [15] utilized the SH index. Pour-Aboughadareh & Poczai [19] constructed a similar venn diagram with these three indices for identifying wheat genotypes while Yue et al. [39] added MTSI index to the diagram for selecting maize genotypes.

### 3.2 Second year selection trial

All of the assessed traits of second-year (FW, RN, RW, MRN, MRW, RL, RD, and DW) displayed statistical significance at a p-value less than 0.05 for the effect of genotype (Table 4), suggesting a significant influence of the genetic makeup of sweet potato genotypes on the examined traits (Supplementary Table S6).

**Table 4:** Analysis of variance of 75 sweet potato genotypes (with four checks) in a RCBD experimental design.

Source of variation	df	MSS							
		FW	RN	RW	MRN	MRW	RL	RD	DW
Replication	2	138148***	13.29*	89623**	7.97^ns^	122393***	28.84**	107.34***	2.32^ns^
Genotypes	74	13457*	6.25***	36630***	13.05*	40021***	8.25**	6.02^ns^	7.74***
Residuals	148	8643	3.40	15703	8.35	15167	5.08	4.65	3.10
CV (%)		29.11	34.59	34.15	70.27	36.63	18.26	12.99	7.51

^df^ degrees of freedom, ^CV^ coefficient of variation, ***significant at p < 0.001, **significant at p < 0.01, *significant at p < 0.05, NS = non-significant, ^FW^ average foliage fresh weight per plant (g), ^RN^ average storage root number per plant, ^RW^ average storage root weight per plant (g), ^MRN^ marketable storage root number per plant, ^MRW^ marketable storage root weight per plant (g), ^RL^ average storage root length (cm), ^RD^ average storage root diameter (cm) and ^DW^ dry weight of storage roots (%).

#### 3.2.1 Factor analysis and selection of genotypes using MGIDI index

The MGIDI index effectively identified five factors by examining the eigenvalue (>1) of principal components (PCs). The cumulative variation observed in these five PCs amounted to 73.2 % (Table 5). In factor analysis (Table 6), the mean of all studied parameters in the chosen genotypes has witnessed an increment, except for RL, which was directed towards the desired objectives. The factor loadings were deemed to be substantial (ranging from 0.48 to 0.92), thus allowing us to infer that the traits were adequately elucidated through factor analysis, and that the selection of genotypes will yield fruitful results. Overall, the chosen genotypes exhibited favorable selection differential values in all traits, excluding RL. The broad-sense heritability was notably high. The average uniqueness of traits amounted to 0.27, with the highest uniqueness (0.66) observed in the RD trait. The selection gain percentage reached its peak in DW, followed by MRW and FW. Chattopadhyay et al. [40] selected the brinjal genotypes based on high heritability and genetic gain.

**Table 5:** Identified principal components (PCs), eigenvalues and their explained variance on MGIDI selection index.

PCs	Eigenvalues	Proportional variance (%)	Cumulative variance (%)
PC1	1.38	17.2	17.2
PC2	1.3	16.3	33.5
PC3	1.11	13.9	47.4
PC4	1.05	13.2	60.6
PC5	1.01	12.6	73.2
Noise	2.14	26.8	–

**Table 6:** Factor analysis with loadings, selection differential (SD), broad-sense heritability (h^2^) and predicted selection gain (SG) based on the MGIDI index for 11 selected sweet potato genotypes.

Traits	Factor	Factor loadings	SD	h^2^	Uniqueness	SG (%)	Sense	Goal
FW	FA4	0.92	15	0.976	0.15	14.3	increase	100
RN	FA5	0.48	7.97	0.981	0.23	7.71	increase	100
RW	FA1	−0.74	1.89	0.979	0.24	1.92	increase	100
MRN	FA2	0.84	6.34	0.98	0.26	5.92	increase	100
MRW	FA1	−0.77	17.1	0.981	0.22	16.4	increase	100
RL	FA5	−0.84	−2.02	0.983	0.21	−1.92	increase	0
RD	FA2	0.53	4.12	0.981	0.66	4.01	increase	100
DW	FA3	0.88	21.1	0.98	0.18	20.4	increase	100
Mean	–	–		–	0.27	–	–	–

^FW^ average foliage fresh weight per plant (g), ^RN^ average storage root number per plant, ^RW^ average storage root weight per plant (g), ^MRN^ marketable storage root number per plant, ^MRW^ marketable storage root weight per plant (g), ^RL^ average storage root length (cm), ^RD^ average storage root diameter (cm) and ^DW^ dry weight of storage roots (%)

**Fig. 2:**
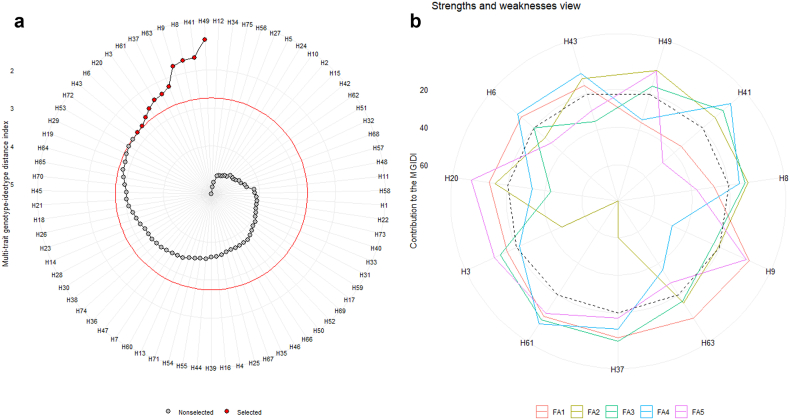
Selection of genotypes through MGIDI index (a) and the strength and weaknesses of selected genotypes (b).

The experimental genotypes are arranged in a descending fashion according to the MGIDI index, with the genotype possessing the highest index value situated at the center, while being placed on the outermost part of the circle that bears the lowest index value (Fig. 2a). The genotypes selected were based on their MGIDI index values, as indicated by the red-colored dots. H49 occupied the top position, with H41, H8, H9, H63, H37, H61, H3, H20, H6, and H43 following suit as the commendable genotypes. Several scholars utilized the MGIDI index as a means of selecting exceptional genotypes, as observed in various crops such as sesame [9], eggplant [41], maize [42] and yam [43].

The strengths and weaknesses of the selected genotypes are illustrated in Fig. 2b. The position (close to center or edge of circle) of factors regarding genotypes represent the respective factors' influence on genotypes, whereas the dotted lines represent the average performance of genotypes in terms of factor contribution. The weakness of each respective genotype is indicated by higher factor values with a tendency to move towards the center, while lower factor values serve as an indication of strength. To illustrate, the genotypes H43, H49, H41, and H8 demonstrated significant strengths as they exhibited the greatest contribution to the second factor (FA2), as opposed to H3, H61, and H37, whose contribution to FA2 was the lowest, thereby highlighting their weaknesses (Fig. 2b). The evaluation of strengths and weaknesses in sweet potato genotypes was effectively conducted through the utilization of the MGIDI index for selection [4].

### 3.3 Third year MET

#### 3.3.1 AMMI analysis

The statistical analysis showed a notable influence of the interaction between genotype and environment on traits such as MRY, DW, BC, and VC at a significance level of p < 0.05 (Table 7). This suggests that there are differences in the average performance of various genotypes and how they interact with different environments (Supplementary Table S7). Conversely, there was a notable effect of the environmental conditions on the traits MRY, DW and VC (Table 7), suggesting that changes in the growing conditions have a significant influence on these particular traits. Therefore, an AMMI analysis was conducted in for identifying the stable genotypes due to the considerable influence of GEI on the parameters under investigation. Two IPCs that are statistically significant in AMMI model, was determined to be the most precise in forecasting the examined traits. Across all the traits, the first principal component accounted for the majority of the observed variation in genotype-environment interaction (Table 7). Specifically, the first IPC explained 85.2 % of the variation in MRY, 92.3 % in DW, 99 % in BC, and 99.3 % in VC. The IPCs were found to be statistically significant at p < 0.05. This particular model emphasizes significant discrepancies in genotypic response across environments, utilizing IPCs. As a result, the AMMI model has been utilized as an effective predictive model for analyzing GEI in diverse crops [[44], [45], [46]]. On the other hand, AMMI lacks the ability to discern the strong correlation between a high average performance and stability [47] and struggles with accommodating a LMM structure [20].

**Table 7:** Additive and multiplicative interaction effect analysis of variance of AMMI model for MRY, DW, BC and VC of 11 sweet potato genotypes.

MRY					
Source of variations	df	MSS	F value	Proportional variance	Cumulative variance
Environment	2	997.84	575.82***	–	–
Error I	6	1.73	0.72^ns^	–	–
Genotype	10	250.08	103.66***	–	–
GEI	20	48.30	20.02***	–	–
PC1	11	68.58	28.43***	78.10	78.10
PC2	9	23.53	9.75***	21.90	100
Error II	60	2.41	–	–	–
Total	118	55.79	–	–	–
DW					
Environment	2	12.27066	5.438277*	–	–
Error I	6	2.25635	0.832957^ns^	–	–
Genotype	10	215.7566	79.64898***	–	–
GEI	20	7.727743	2.852783***	–	–
PC1	11	12.96968	4.79***	92.3	92.3
PC2	9	1.32093	0.49^ns^	7.7	100
Error II	60	2.708844	–	–	–
Total	118	22.60412	–	–	–
BC					
Environment	2	3.698079	0.464182^ns^	–	–
Error I	6	7.966866	1.811431^ns^	–	–
Genotype	10	982.9968	223.5046***	–	–
GEI	20	26.88329	6.112471***	–	–
PC1	11	48.38155	11***	99	99
PC2	9	0.60764	0.14^ns^	1	100
Error II	60	4.398105	–	–	–
Total	118	95.12189	–	–	–
VC					
Environment	2	9.334455	6.633173*	–	–
Error I	6	1.407238	0.411242^ns^	–	–
Genotype	10	222.2501	64.94883***	–	–
GEI	20	12.99844	3.798574***	–	–
PC1	11	23.45776	6.86***	99.3	99.3
PC2	9	0.21482	0.06^ns^	0.7	100
Error II	60	3.421926	–	–	–
Total	118	25.21073	–	–	–

***significant at p < 0.001, ** significant at p < 0.01, * significant at p < 0.05, ^NS^ non-significant, ^MRY^ marketable storage root yield (t/ha), ^DW^ dry weight of storage roots (%), ^BC^ beta-carotene content (mg/100g) and ^VC^ vitamin C content (mg/100g).

#### 3.3.2 Variance estimation of genetic parameters

The effect of genotype and GEI on the studied traits was found significant at p < 0.05 based on likelihood ratio test (Table 8). In this case, the utilization of BLUP method over AMMI can yield superior outcomes [20]. The results revealed high heritability values (0.56, 0.84, 0.90, and 0.78) for MRY, DW, BC, and VC, respectively (Table 8). The estimation of heritability in traits plays a crucial role in breeding programs by identifying and recommending superior genotypes [48]. The GEI correlation coefficient was low for yield and quality parameters of 11 sweet potato genotypes. The accuracy of genotype selection demonstrated high values ranging from 0.90 to 0.99 (Table 8) signifies the relationship between the values of observed and predicted [20]. The high values of selection accuracy observed for the studied parameters indicate the model reliability for the superior genotype selection [29].

**Table 8:** Variance components estimation from a linear mixed model (LMMs) for MRY, DW, BC and VC in 11 sweet potato genotypes.

Parameters	MRY	DW	BC	VC
Genotype	22.42**	23.11***	106.2***	23.25***
GEI	15.3***	1.673**	7.092***	3.192***
Residual	2.41	2.71	4.62	3.42
Heritability	0.56	0.84	0.90	0.78
r^2^(GEI)	0.38	0.06	0.06	0.11
Accuracy	0.90	0.98	0.99	0.97

***significant at p < 0.001, ** significant at p < 0.01, ^MRY^ marketable storage root yield (t/ha), ^DW^ dry weight of storage roots (%), ^BC^ beta-carotene content (mg/100g), ^VC^ vitamin C content (mg/100g) and ^r2(GEI)^ GEI correlation coefficient.

#### 3.3.3 Predicted mean value estimation using BLUP

**Fig. 3:**
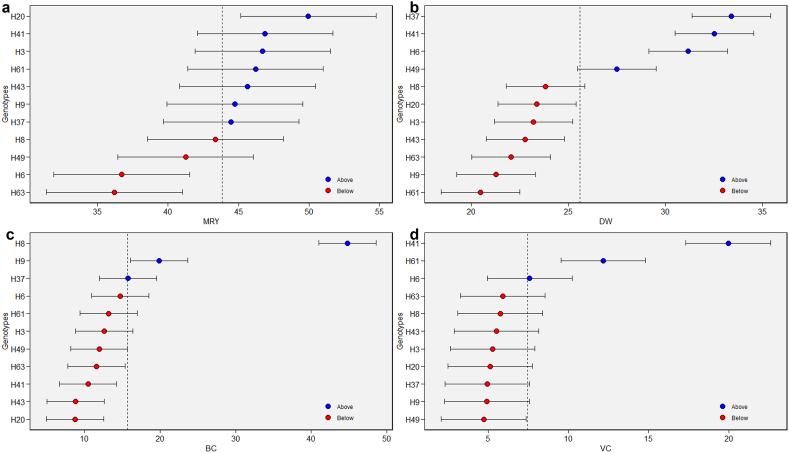
BLUP value for MRY (a), DW (b), BC (c) and VC (d). ^MRY^ marketable storage root yield (t/ha), ^DW^ dry weight of storage roots (%), ^BC^ beta-carotene content (mg/100g) and ^VC^ vitamin C content (mg/100g).

The predicted mean values of genotypes for MRY, DW, BC, and VC as indicated in Fig. 3. Among the genotypes, seven for MRY, four for DW, three for BC, and VC exhibited higher than predicted mean value. H20 demonstrated the highest predicted mean value for MRY, followed by H41, H3, H61, H43, H9, and H37 (Fig. 3a). In the case of DW, H37 displayed the highest predicted mean value, followed by H41, H6, and H49 (Fig. 3b). Genotype H8 exhibited the highest predicted mean value for BC, followed by H9 and H37 (Fig. 3c). The predicted mean value for VC was highest in genotype H41, followed by H61 and H6 (Fig. 3d). The genotypes' predicted average values were deemed suitable for the parameters under study. The BLUP method, renowned for its remarkable efficacy, provides estimations regarding the mean genotypic performances within mixed models. Thus, it can bridge the gap in the analysis of LMM structure left by AMMI.

Several scholars have employed the BLUP in the evaluation of various sweet potato varieties, yielding useful results [23,24,[49], [50], [51]]. The accurate estimation of the mean performance of genotypes, particularly in LMMs, is one of the significant benefits of the BLUP method [20]. Moreover, in cases where an LMM effect is present in the findings, the BLUP allows for the most accurate forecasting of random effects [29]. However, there is a current demand for effectively handle and represent a random GEI structure through graphical means. The estimates through BLUP, may not fully encompass the complexities and intricacies of a random GEI structure.

#### 3.3.4 Stability analysis using WAAS model

**Fig. 4:**
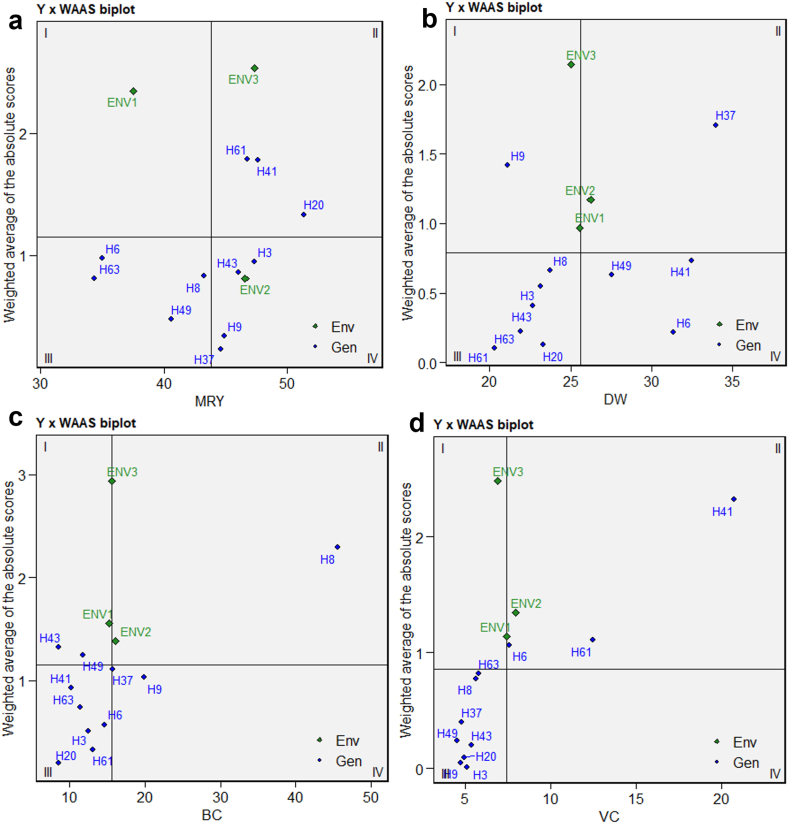
WAAS biplot of AMMI stability model with mean performance of MRY (a), DW (b), BC (c) and VC (d). ^MRY^ marketable storage root yield (t/ha), ^DW^ dry weight of storage roots (%), ^BC^ beta-carotene content (mg/100g) and ^VC^ vitamin C content (mg/100g).

The matrix of BLUPs for the GEI effects generated by an LMM is referred to as WAAS, which stands for the singular value decomposition. The WAAS biplot, depicted in Fig. 4, differs from the AMMI model in that it incorporates all scores of the principal components (PCs) rather than solely considering the first PC. The WAAS biplot, as a consequence, offers an assessment of stability by considering the entirety of the GEI variance in the identification of genotypes' stability. The centrally positioned vertical line serves to exhibit the aggregate average of MRY (Fig. 4a), DW (Fig. 4b), BC (Fig. 4c), and VC (Fig. 4d) across the three experimental environments. The position status to the right of central vertical line exhibits a higher yield compared to the overall mean value, while those on the left side demonstrate a lower yield value in relation to the studied trait's overall mean. The mean of the WAAS is represented by the horizontal axis, which is positioned in the middle of the biplot. The biplot is partitioned into four quadrants, predicated on the location at which this axis intersects with the vertical axis. Genotypes situated within distinct quadrants possess the ability to be categorized based on their adaptability towards diverse environments. For MRY and VC, no genotypes were found in the first quadrant. H9 was associated with DW, while H43 and H49 were associated with BC, yielding lower values than the overall mean and exhibiting varied WAAS values. This implies that these genotypes exhibit a notable variation and lack stability in relation to the specific trait across various environments, resulting in a performance measure that is below the mean. Generally speaking, it is not recommended to cultivate these genotypes. Genotypes H61 and H41, which are associated with MRY, H37 for DW, H8 related to BC, and H41 for VC, are positioned in the second quadrant. These genotypes exhibit high WAAS and performance values that exceed the overall average. If the environmental circumstances are advantageous, the productivity of these genotypes will be substantial, rendering them suitable for cultivation in regions that exhibit optimal conditions for the yield and quality of sweet potatoes. In this scenario, genotype H20, which is related to MRY, and genotypes H6 and H61, which are associated with VC and located in the second quadrant, are considered stable. Genotypes H6 and H63, linked to MRY, H8 and H3, associated with DW, and H41 and H63, related to BC, as well as H63, H8, and H37, related to VC and positioned in the third quarter, exhibit lower WAAS. This suggests that they are stable or less influenced by environmental conditions. Moreover, these genotypes show lower performance values. Genotypes H3 and H43, linked to MRY, H41 and H49, associated with DW, and H37 and H9, related to BC, positioned in the fourth quadrant of the biplot, have lower WAAS and higher performance values compared to the overall average. Genotypes situated in fourth quadrant are stable and exhibiting superior performances owing to their limited impact on environment and satisfactory performance. When examining the WAAS biplot, genotypes with WAAS values of zero or near zero are considered the most stable. Conversely, the genotypes that exhibit a yield value exceeding the mean are regarded as the most desirable. As a result, it can be asserted that H20 and H3, in terms of MRY, as well as H41 and H49, in terms of DW, and furthermore H37 and H9, in terms of BC, and lastly, the genotype H6 and H61, in terms of VC, endorse a low level of GEI and a high level of stability. However, when considering the desired WAAS value, the ideal genotypes have yield surpasses the overall average. Thus, it is evident that genotypes H20, in terms of MRY, H41 for DW, H9 for BC, and H61 for VC, not only demonstrate stability but also possess a yield value that exceeds the overall average. Consequently, it is with great discernment that these genotypes are selected as stable genotypes with superior performance. Several studies conducted based on WAAS index to identify stable genotypes [29,52,53].

#### 3.3.5 Stability analysis using WAASBY model

**Fig. 5:**
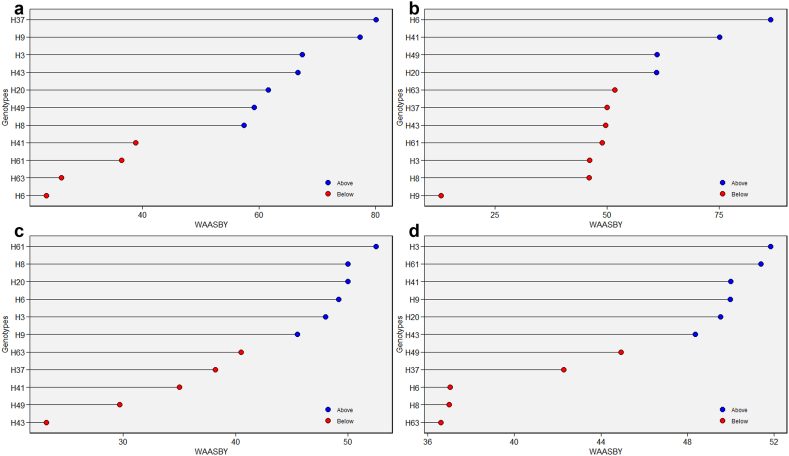
WAASBY index value for trait MRY (a), DW (b), BC (c) and VC (d). ^MRY^ marketable storage root yield (t/ha), ^DW^ dry weight of storage roots (%), ^BC^ beta-carotene content (mg/100g) and ^VC^ vitamin C content (mg/100g).

In Fig. 5, the possibility arises to rank and choose genotypes simultaneously based on the yield and stability value. The utilization of the WASSBY, which is the amalgamation of the WAASB and the yield, is employed with the intention of achieving both high yield and stability. Higher than average WAASBY index is indicated by blue circles, while lower than average WAASBY index is shown by red circles. In terms of MRY, H37 and H9 exhibited a WAASBY index that was higher than the mean, followed by H3, H43, H20, H49, and H8 when compared to other genotypes (Fig. 5a). Based on WAASBY, H6, H41, H49, and H20 were identified as stable genotypes with high DW (Fig. 5b). Similarly, genotype H61, H8, H20, H6, H3, and H9 were selected as the most optimal genotype with stability in relation to BC (Fig. 5c). Genotype H3, H61, H41, H9, H20, and H43 were chosen as stable and superior in terms of VC (Fig. 5d). Stability parameters play a pivotal role in the field of plant breeding as they hold an utmost importance in securing the uniformity and constancy of different traits across diverse environmental settings and external factors. The WAASBY index comprehensively considers both the stability factors and performance traits simultaneously [20].

#### 3.3.6 GGE polygonal biplot analysis for MET

**Fig. 6:**
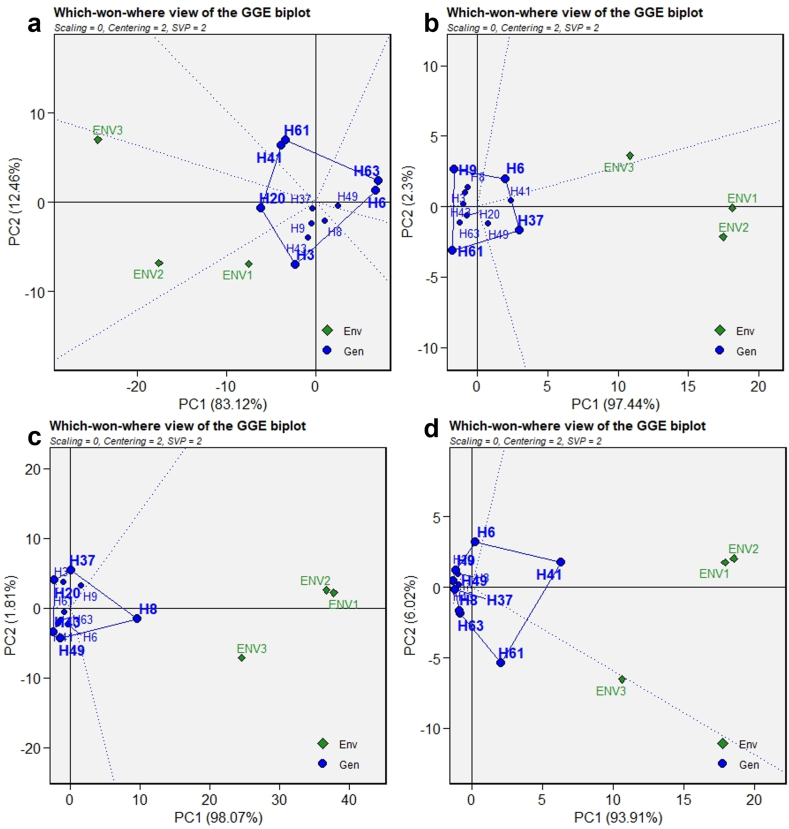
Polygonal view of GGE biplot for winner genotype in respective environment for MRY (a), DW (b), BC (c) and VC (d). ^MRY^ marketable storage root yield (t/ha), ^DW^ dry weight of storage roots (%), ^BC^ beta-carotene content (mg/100g) and ^VC^ vitamin C content (mg/100g).

The purpose of GGE polygonal biplot (Fig. 6) is to discern the MEs and superior genotypes in distinct environments. Within this biplot, a polygon is constructed by connecting genotypes that exhibit the greatest deviation from the coordinate origin. Genotypes H63, H6, H3, H20, H41 and H61 in Fig. 6a, H6, H37, H61 and H9 in Fig. 6b, H37, H8, H49, H43 and H20 in Fig. 6c, and H41, H61, H63, H8, H37, H49, H9 and H6 in Fig. 6d were farthest from origin and formed respective polygon for each trait. From the point of origin in the coordinate system, vertical lines were extended along the sides of the given polygon, thereby establishing the respective MEs [3]. In the regions where the environments were situated and genotypes were present above them, it signifies that these particular genotypes exhibit optimal performance within those specific environments. Genotype H20 displayed superior performance in ENV2 and ENV3, while H3 showed the highest performance in ENV1 in relation to MRY. As for DW, H37 exhibited the best and most stable performance in ENV1 and ENV2, while H6 demonstrated the same in ENV3. In all environments studied, Genotype H8 displayed the highest BC. In terms of VC, H41 exhibited the best and stable performance in ENV1 and ENV2, while H61 emerged as the top performer in ENV3. Genotypes that are situated in areas devoid of any environments are deemed unsuitable for cultivation in any of the examined environments and are classified as feeble genotypes in the majority of the environments. Using a polygonal biplot, researchers effectively selected elite sweet potato genotypes that demonstrate the highest adaptability to specific environments with respective trait of interest [3,[54], [55], [56]]. Therefore, it is crucial to implement proactive measures for developing sweet potato varieties that are stable and adaptable to target environments.

#### 3.3.7 Multitrait stability index (MTSI)

The selected genotypes’ mean value for MRY has witnessed an increase, aligning with the intended objectives (Table 9). Overall, the selected genotypes have led to a negative selection differential for all studied parameters, except for MRY. Nonetheless, these traits exhibited stability and possessed a high heritability (0.81–0.97). Furthermore, a few genotypes demonstrated exceptional performance in the case of traits DW, BC, and VC. For instance, H8 excelled in terms of BC, as elucidated in the BLUP model (Fig. 3), WAAS biplot (Fig. 4), and GGE biplot (Fig. 6). Consequently, the mean value of certain traits reached a high point, and the selection differential turned negative for those traits. Several researchers have utilized MTSI index for selecting stable genotypes [29,34,57,58]. These findings are in accordance with the outcomes attained from our investigation, thereby emphasizing the efficacy of MTSI in discerning superior genotypes.

**Table 9:** Factor analysis, selection differential (SD), broad-sense heritability (h^2^) and selection gain (SG) (%) for MRY, DW, BC and VC of 11 sweet potato genotypes during indexing of MTSI.

Traits	Factors	$\bar{X}$_o_	$\bar{X}$_s_	SD	h^2^	SG (%)	Sense	Goal
DW	FA1	1	25.6	−2.39	0.964	−8.99	increase	0
BC	FA1	1	15.7	−5.13	0.974	−31.8	increase	0
MRY	FA2	2	43.8	5.57	0.807	10.3	increase	100
VC	FA2	2	7.43	−2.39	0.942	−30.2	increase	0

$\bar{X}$_o_ = observed mean, $\bar{X}$_s_ = predicted mean.

**Fig. 7:**
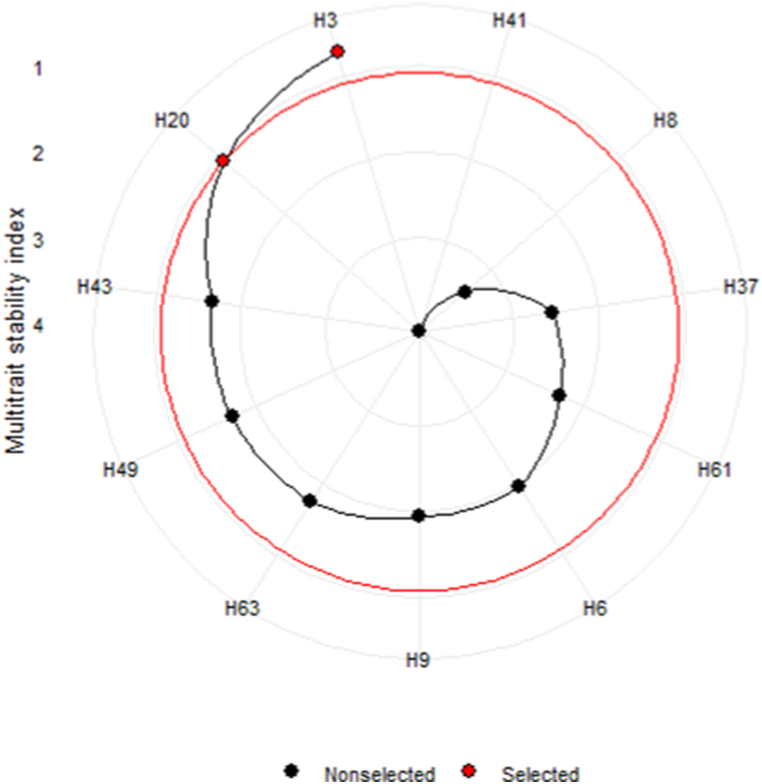
Ranking of genotype stability based on MTSI index.

MTSI was calculated based on four attributes pertaining to yield and quality. In Fig. 7, the experimental genotypes are arranged based on their MTSI values, sorted in descending order. The genotype with the highest MTSI value is placed in the center, while the genotype with the lowest value is positioned in the outermost circle, representing its high stability rank. The red dot genotypes were selected through MTSI index. Based on MTSI index, H3 occupied the top ranked stable genotype, followed by H20. The high yielding and stable sweet potato genotypes regarding multi traits were selected using MTSI index [5].

## 4 Conclusion

The multi-year assessment trials and stability examination of sweet potato collections have yielded valuable insights regarding the diversity, heritability, and performance of these genotypes. The examination of variance has unveiled notable deviations among the genotypes with respect to diverse agronomic features linked to yield. The process of selection, which is grounded on diversified indices such as MGIDI, SH, and FAI-BLUP, has ultimately resulted in the identification of high-performance genotypes. The analysis of AMMI and the estimation facilitated by LMM have allowed for the evaluation of GEIs and heritability, thereby further aiding in the selection of genotypes. The comprehensive assessment of genotype stability and adaptability across distinct environments has been accomplished through the implementation of WAAS and WAASBY stability analyses, in conjunction with the GGE polygonal biplot. The MTSI index has emerged as a robust tool for the identification of genotypes that exhibit high stability and performance across multiple traits. In summary, this study offers an extensive framework for the selection of superior sweet potato genotypes with stability, high yield, and quality, thereby contributing to the enhancement of sweet potato breeding programs.

## Data availability

Data will be made available on request.

## CRediT authorship contribution statement

**Zakaria Alam:** Writing – review & editing, Writing – original draft, Supervision, Methodology, Investigation, Formal analysis, Data curation, Conceptualization. **Sanjida Akter:** Formal analysis, Data curation. **Md Anwar Hossain Khan:** Supervision, Investigation. **Md Iqbal Hossain:** Formal analysis, Data curation. **Md Nurul Amin:** Formal analysis, Data curation. **Avijit Biswas:** Formal analysis, Data curation. **Ebna Habib Md Shofiur Rahaman:** Investigation, Data curation. **Mir Aszad Ali:** Investigation, Data curation. **Debashish Chanda:** Investigation, Data curation. **Md Hasan Sofiur Rahman:** Formal analysis, Data curation. **Md Abu Kawochar:** Formal analysis, Data curation. **Md Shamshul Alam:** Supervision, Data curation, Conceptualization. **Mohammad Mainuddin Molla:** Methodology, Data curation, Conceptualization. **Md Monirul Islam:** Data curation, Conceptualization. **M.A.H.S. Jahan:** Investigation, Conceptualization. **Md Zulfikar Haider Prodhan:** Methodology, Investigation, Conceptualization. **Md Monjurul Kadir:** Investigation, Formal analysis, Data curation. **Debasish Sarker:** Resources, Methodology, Conceptualization.

## Declaration of competing interest

The authors declare the following financial interests/personal relationships which may be considered as potential competing interests. Zakaria Alam reports financial support, administrative support, and equipment, drugs, or supplies were provided by 10.13039/501100005867Bangladesh Agricultural Research Institute, Bangladesh. The International Potato Centre (CIP) in Lima, Peru, arranged the article processing charge (APC) for this manuscript.
