# Supplementary material for: Sweet potato (Ipomoea batatas L.) genotype selection using advanced indices and statistical models: A multi-year approach
Source: Heliyon. 2024 May 20;10(10):e31569. doi: 10.1016/j.heliyon.2024.e31569 (PMC11141454; doi:10.1016/j.heliyon.2024.e31569)
Supplement: Multimedia component 1 [file mmc1.docx]

Supplementary Table S1. Collected 351 sweet potato genotypes including four checks with their country of collection

| Genotype | Source | Genotype | Source | Genotype | Source | Genotype | Source | Genotype | Source | Genotype | Source | Genotype | Source |
| --- | --- | --- | --- | --- | --- | --- | --- | --- | --- | --- | --- | --- | --- |
| BARIMistialu-8 | Bangladesh | Deshisada2/12 | Bangladesh | H16.ej.10 | Bangladesh | Indo7 | Indonesia | JPN28 | Japan | Moz22 | Mozambique | Moz2.48 | Mozambique |
| BARIMistialu-12 | Bangladesh | H2.34/11 | Bangladesh | H2.21/11.24 | Bangladesh | Indo8 | Indonesia | JPN29 | Japan | Moz23 | Mozambique | Moz2.49 | Mozambique |
| BARIMistialu-14 | Bangladesh | H9.13 | Bangladesh | H2.21/11.25 | Bangladesh | Indo9 | Indonesia | JPN30 | Japan | Moz24 | Mozambique | Moz2.50 | Mozambique |
| BARIMistialu-15 | Bangladesh | Deshisada3/14 | Bangladesh | H2.21/11.26 | Bangladesh | Indo10 | Indonesia | JPN31 | Japan | Moz25 | Mozambique | Moz2.51 | Mozambique |
| Deshisada1/19 | Bangladesh | H2.21/11 | Bangladesh | H9.10.1 | Bangladesh | Indo11 | Indonesia | JPN32 | Japan | Moz2.2 | Mozambique | Moz2.52 | Mozambique |
| H4.2/12 | Bangladesh | H11.15/10 | Bangladesh | H9.10.2 | Bangladesh | Indo12 | Indonesia | JPN33 | Japan | Moz2.3 | Mozambique | Moz2.53 | Mozambique |
| H3.22/31 | Bangladesh | H9.32/11 | Bangladesh | H9.10.3 | Bangladesh | Indo13 | Indonesia | JPN34 | Japan | Moz2.4 | Mozambique | Moz2.54 | Mozambique |
| H3.22/32 | Bangladesh | H2.12.11 | Bangladesh | H9.10.4 | Bangladesh | Indo14 | Indonesia | JPN35 | Japan | Moz2.5 | Mozambique | Moz2.55 | Mozambique |
| H3.22/33 | Bangladesh | H5.3/12.1 | Bangladesh | H9.10.5 | Bangladesh | Indo15 | Indonesia | JPN36 | Japan | Moz2.6 | Mozambique | Moz2.56 | Mozambique |
| H3.22/34 | Bangladesh | H5.3/12.2 | Bangladesh | H9.10.6 | Bangladesh | Indo16 | Indonesia | JPN37 | Japan | Moz2.7 | Mozambique | Moz2.57 | Mozambique |
| H3.22/35 | Bangladesh | H5.3/12.3 | Bangladesh | H9.10.7 | Bangladesh | Indo17 | Indonesia | JPN38 | Japan | Moz2.8 | Mozambique | Moz2.58 | Mozambique |
| H3.22/36 | Bangladesh | H5.3/12.4 | Bangladesh | H199024.39/11 | Bangladesh | Indo18 | Indonesia | JPN39 | Japan | Moz2.9 | Mozambique | Moz2.59 | Mozambique |
| H3.22/37 | Bangladesh | H5.3/12.5 | Bangladesh | H5.14/09.30 | Bangladesh | Indo19 | Indonesia | JPN40 | Japan | Moz2.10 | Mozambique | Moz2.60 | Mozambique |
| H3.22/38 | Bangladesh | H5.3/12.6 | Bangladesh | H9.10.9 | Bangladesh | Indo20 | Indonesia | JPN41 | Japan | Moz2.11 | Mozambique | Moz2.61 | Mozambique |
| H3.22/39 | Bangladesh | H5.3/12.7 | Bangladesh | H9.10.10 | Bangladesh | Indo21 | Indonesia | JPN42 | Japan | Moz2.12 | Mozambique | Moz1.1 | Mozambique |
| H3.22/40 | Bangladesh | H5.3/12.8 | Bangladesh | H9.10.11 | Bangladesh | Indo22 | Indonesia | JPN43 | Japan | Moz2.13 | Mozambique | Moz1.2 | Mozambique |
| H6.19/12 | Bangladesh | H11.20/15 | Bangladesh | H9.10.12 | Bangladesh | Indo23 | Indonesia | JPN44 | Japan | Moz2.14 | Mozambique | Moz1.3 | Mozambique |
| H5.14/09.1 | Bangladesh | H5.3/12.9 | Bangladesh | H3.22/6 | Bangladesh | Indo24 | Indonesia | JPN45 | Japan | Moz2.15 | Mozambique | Moz1.4 | Mozambique |
| H5.14/09.2 | Bangladesh | H5.3/12.10 | Bangladesh | H3.22/7 | Bangladesh | Indo25 | Indonesia | JPN46 | Japan | Moz2.16 | Mozambique | Moz1.5 | Mozambique |
| H5.14/09.3 | Bangladesh | H5.3/12.11 | Bangladesh | H3.22/8 | Bangladesh | Indo26 | Indonesia | JPN47 | Japan | Moz2.17 | Mozambique | Moz1.6 | Mozambique |
| H5.14/09.4 | Bangladesh | H5.ej.10 | Bangladesh | H3.22/9 | Bangladesh | Indo27 | Indonesia | JPN48 | Japan | Moz2.18 | Mozambique | Moz1.7 | Mozambique |
| H5.14/09.5 | Bangladesh | H5.3/12.12 | Bangladesh | H3.22/10 | Bangladesh | Indo28 | Indonesia | JPN49 | Japan | Moz2.19 | Mozambique | Moz1.8 | Mozambique |
| H5.14/09.6 | Bangladesh | H5.3/12.13 | Bangladesh | H9.7.12 | Bangladesh | JPN1.1144.2 | Japan | JPN50 | Japan | Moz2.20 | Mozambique | Moz1.10 | Mozambique |
| H5.14/09.7 | Bangladesh | H5.3/12.14 | Bangladesh | H3.22/11 | Bangladesh | JPN9 | Japan | JPN51 | Japan | Moz2.21 | Mozambique | Moz1.11 | Mozambique |
| H5.14/09.8 | Bangladesh | H5.3/12.15 | Bangladesh | H3.22/12 | Bangladesh | JPN2 | Japan | JPN52 | Japan | Moz2.22 | Mozambique | Moz1.12 | Mozambique |
| H5.14/09.9 | Bangladesh | H2.21/11.1 | Bangladesh | H3.22/13 | Bangladesh | JPN1 | Japan | JPN53 | Japan | Moz2.23 | Mozambique | Moz1.13 | Mozambique |
| H5.14/09.10 | Bangladesh | H2.21/11.2 | Bangladesh | H3.22/14 | Bangladesh | JPN3 | Japan | JPN55 | Japan | Moz2.24 | Mozambique | Moz1.14 | Mozambique |
| H6.3/14 | Bangladesh | H2.21/11.3 | Bangladesh | H3.22/15 | Bangladesh | JPN4 | Japan | JPN56 | Japan | Moz2.25 | Mozambique | Moz1.16 | Mozambique |
| H5.14/09.11 | Bangladesh | H9.48.11 | Bangladesh | H3.22/16 | Bangladesh | SPO104 | Japan | JPN5 | Japan | Moz2.26 | Mozambique | Moz1.17 | Mozambique |
| H5.14/09.12 | Bangladesh | H2.21/11.4 | Bangladesh | H3.22/17 | Bangladesh | JPN6 | Japan | Moz1 | Mozambique | Moz2.27 | Mozambique | Moz1.18 | Mozambique |
| H5.14/09.13 | Bangladesh | H2.21/11.5 | Bangladesh | H3.22/18 | Bangladesh | JPN7 | Japan | Moz2 | Mozambique | Moz2.28 | Mozambique | Moz1.19 | Mozambique |
| H5.14/09.14 | Bangladesh | H2.21/11.6 | Bangladesh | H3.22/19 | Bangladesh | JPN8 | Japan | Moz3 | Mozambique | Moz2.29 | Mozambique | Moz1.20 | Mozambique |
| H5.14/09.15 | Bangladesh | H2.21/11.7 | Bangladesh | H3.22/20 | Bangladesh | JPN10 | Japan | Moz4 | Mozambique | Moz2.30 | Mozambique | Moz1.21 | Mozambique |
| H5.14/09.16 | Bangladesh | H2.21/11.8 | Bangladesh | H27.2/12 | Bangladesh | JPN11 | Japan | Moz5 | Mozambique | Moz2.31 | Mozambique | Moz1.22 | Mozambique |
| H5.14/09.17 | Bangladesh | H2.21/11.9 | Bangladesh | H3.22/21 | Bangladesh | JPN12 | Japan | Moz6 | Mozambique | Moz2.32 | Mozambique | Moz1.23 | Mozambique |
| H5.14/09.18 | Bangladesh | H2.21/11.10 | Bangladesh | H3.22/22 | Bangladesh | JPN13 | Japan | Moz7 | Mozambique | Moz2.33 | Mozambique | Moz1.24 | Mozambique |
| H5.14/09.19 | Bangladesh | H2.21/11.11 | Bangladesh | H3.22/23 | Bangladesh | JPN14 | Japan | Moz8 | Mozambique | Moz2.34 | Mozambique | Moz1.25 | Mozambique |
| H5.14/09.20 | Bangladesh | H2.21/11.12 | Bangladesh | H3.22/24 | Bangladesh | JPN15 | Japan | Moz9 | Mozambique | Moz2.35 | Mozambique | Moz1.26 | Mozambique |
| H9.6/11 | Bangladesh | H2.21/11.13 | Bangladesh | H3.22/25 | Bangladesh | JPN16 | Japan | Moz10 | Mozambique | Moz2.36 | Mozambique | Moz1.27 | Mozambique |
| H5.14/09.21 | Bangladesh | H199024.22/11 | Bangladesh | H3.22/26 | Bangladesh | JPN17 | Japan | Moz11 | Mozambique | Moz2.37 | Mozambique | Moz1.28 | Mozambique |
| H5.14/09.22 | Bangladesh | H2.21/11.14 | Bangladesh | H3.22/27 | Bangladesh | JPN18 | Japan | Moz12 | Mozambique | Moz2.38 | Mozambique | Moz1.29 | Mozambique |
| H5.14/09.23 | Bangladesh | H2.21/11.15 | Bangladesh | H3.22/28 | Bangladesh | JPN19 | Japan | Moz13 | Mozambique | Moz2.39 | Mozambique | Moz1.30 | Mozambique |
| H5.14/09.24 | Bangladesh | H2.21/11.16 | Bangladesh | H3.22/29 | Bangladesh | JPN20 | Japan | Moz14 | Mozambique | Moz2.40 | Mozambique | Moz1.31 | Mozambique |
| H5.14/09.25 | Bangladesh | H2.21/11.17 | Bangladesh | H3.22/30 | Bangladesh | JPN21 | Japan | Moz15 | Mozambique | Moz2.41 | Mozambique | Moz1.32 | Mozambique |
| H5.14/09.26 | Bangladesh | H2.21/11.18 | Bangladesh | Indo1 | Indonesia | JPN22 | Japan | Moz16 | Mozambique | Moz2.42 | Mozambique | Moz1.33 | Mozambique |
| H5.14/09.27 | Bangladesh | H2.21/11.19 | Bangladesh | Indo2 | Indonesia | JPN23 | Japan | Moz17 | Mozambique | Moz2.43 | Mozambique | Moz1.34 | Mozambique |
| H5.14/09.28 | Bangladesh | H2.21/11.20 | Bangladesh | Indo3 | Indonesia | JPN24 | Japan | Moz18 | Mozambique | Moz2.44 | Mozambique | Moz1.35 | Mozambique |
| H5.14/09.29 | Bangladesh | H2.21/11.21 | Bangladesh | Indo4 | Indonesia | JPN25 | Japan | Moz19 | Mozambique | Moz2.45 | Mozambique | Moz1.36 | Mozambique |
| H6.52.11 | Bangladesh | H2.21/11.22 | Bangladesh | Indo5 | Indonesia | JPN26 | Japan | Moz20 | Mozambique | Moz2.46 | Mozambique | Moz1.37 | Mozambique |
| H9.9/12 | Bangladesh | H2.21/11.23 | Bangladesh | Indo6 | Indonesia | SPM103 | Japan | Moz21 | Mozambique | Moz2.47 | Mozambique | Moz1.38 | Mozambique |
| Moz1.9 | Mozambique | Moz1.15 | Mozambique | Moz1.41 | Mozambique | Moz1.40 | Mozambique | Moz1.39 | Mozambique |  |  |  |  |

Supplementary Table S2. A comprehensive overview of 351 collected sweet potato genotypes with detailed climate description of the source of collections and codes used in the study for graphical illustration

| Country of collection | Number of collected genotypes | Altitude (m) | Average rainfall (mm) | Average temperature (⁰C) | Humidity (%) | Average sunshine (h) |
| --- | --- | --- | --- | --- | --- | --- |
| Mozambique | 126 | 345 | 109.73 | 25.62 | 71.59 | 10.59 |
| Japan | 57 | 438 | 84.47 | 15.19 | 76.09 | 9.92 |
| Indonesia | 28 | 367 | 136.87 | 27.11 | 79.69 | 10.56 |
| Bangladesh | ^140+4CK^ | 105 | 102 | 27.74 | 66.29 | 10.65 |

^+4CK^check varieties

Supplementary Table S3. Monthly mean temperature, precipitation, humidity and sunshine statistics for three environments: Gazipur, Bogura and Jamalpur

| Environment (ID) | | | | | | | | | | | | |
| --- | --- | --- | --- | --- | --- | --- | --- | --- | --- | --- | --- | --- |
| Months | Gazipur (ENV1) | | | | Bogura (ENV2) | | | | Jamalpur (ENV3) | | | |
|  | Temperature (⁰C) | Precipitation (mm) | Humidity (%) | Sunshine (h) | Temperature (⁰C) | Precipitation (mm) | Humidity (%) | Sunshine (h) | Temperature (⁰C) | Precipitation (mm) | Humidity (%) | Sunshine (h) |
| November | 26.34 | 17.44 | 67.15 | 8.78 | 26.94 | 2.35 | 62.2 | 11.34 | 25 | 16.55 | 63.71 | 8.33 |
| December | 23.11 | 5.29 | 60.76 | 8.8 | 23.39 | 1.94 | 54.48 | 9.12 | 21.93 | 5.02 | 57.66 | 8.35 |
| January | 21.89 | 1.34 | 53.97 | 8.9 | 21.85 | 2.98 | 51.88 | 9.16 | 20.77 | 1.27 | 51.21 | 8.45 |
| February | 25.27 | 11.67 | 46.82 | 9.08 | 25.97 | 9.96 | 43.46 | 9.3 | 23.98 | 11.07 | 44.43 | 8.61 |
| March | 29.83 | 23.63 | 49.66 | 11.98 | 31.58 | 9.7 | 37.1 | 12.09 | 28.31 | 22.42 | 47.12 | 11.37 |

Supplementary Table S4: Physico-chemical characteristics of the soil in the research area

| Properties | Value | | |
| --- | --- | --- | --- |
|  | Location | | |
|  | Gazipur | Bogura | Jamalpur |
| Texture | Silty clay loam | Sandy loam | Sandy loam |
| pH (H_2_O) | 5.73 | 6.23 | 6.48 |
| Organic matter (%) | 1.75 | 3.50 | 1.42 |
| Total nitrogen (%) | 0.05 | 0.10 | 0.08 |
| Available phosphorus (ppm) | 35 | 28 | 12.85 |
| Exchangeable Ca (meq/100 g) | 1.30 | 0.16 | 2.50 |
| Exchangeable K (meq/100 g) | 0.71 | 0.60 | 0.09 |
| Exchangeable Mg (meq/100 g) | 0.39 | 0.10 | 0.08 |
| Exchangeable Na (meq/100 g) | 1.22 | 1.52 | 0.31 |
| Exchangeable Fe (meq/100 g) | 31 | 28 | 35.42 |
| Exchangeable Mn (meq/100 g) | 7.2 | 6.32 | 15.23 |
| Exchangeable Cu (meq/100 g) | 0.51 | 0.23 | 1.02 |
| Exchangeable Zn (meq/100 g) | 0.80 | 0.74 | 1.53 |

Supplementary Table S5. Adjusted mean values for yield and yield contributing traits of 351 sweet potato genotypes, along with four check varieties, in an augmented experimental design at Bogura, Bangladesh during the first season

| Genotypes | FW | RN | RW | MRN | MRW |
| --- | --- | --- | --- | --- | --- |
| BARIMistialu-12^CK^ | 310.52 | 7.29 | 767.83 | 4.25 | 621.92 |
| BARIMistialu-14^CK^ | 327.22 | 6.67 | 621.21 | 3.79 | 495.14 |
| BARIMistialu-15^CK^ | 478.18 | 8.37 | 654.74 | 3.46 | 459.20 |
| BARIMistialu-8^CK^ | 536.75 | 6.02 | 862.61 | 3.31 | 690.53 |
| Deshisada1/19 | 306.84 | 5.91 | 662.03 | 2.92 | 438.51 |
| Deshisada2/12 | 304.27 | 6.64 | 291.57 | 1.53 | 108.97 |
| Deshisada3/14 | 514.91 | 5.68 | 1000.87 | 4.70 | 922.75 |
| H11.15/10 | 174.44 | 7.91 | 540.78 | 2.82 | 352.51 |
| H11.20/15 | 517.84 | 8.16 | 675.53 | 3.42 | 412.76 |
| H16.ej.10 | 516.92 | 10.43 | 811.09 | 4.05 | 463.95 |
| H199024.22/11 | 603.25 | 4.10 | 356.42 | 2.05 | 261.95 |
| H199024.39/11 | 605.84 | 3.91 | 696.78 | 4.42 | 715.51 |
| H2.12.11 | 69.24 | 6.68 | 523.54 | 3.53 | 355.42 |
| H2.21/11 | -94.09 | -0.32 | -36.13 | 0.20 | -17.25 |
| H2.21/11.1 | 681.61 | 7.64 | 524.90 | 2.19 | 220.63 |
| H2.21/11.10 | 354.94 | 8.11 | 737.10 | 3.66 | 513.10 |
| H2.21/11.11 | 115.76 | -0.34 | 241.79 | 0.06 | 189.27 |
| H2.21/11.12 | -17.06 | -0.69 | -156.10 | -0.14 | -126.70 |
| H2.21/11.13 | 861.94 | 10.31 | 647.90 | 4.86 | 379.30 |
| H2.21/11.14 | -17.06 | -0.69 | -156.10 | -0.14 | -126.70 |
| H2.21/11.15 | 957.27 | 9.98 | 818.23 | 2.86 | 453.97 |
| H2.21/11.16 | 457.94 | 4.81 | 329.90 | 1.36 | 146.30 |
| H2.21/11.17 | -17.06 | -0.69 | -156.10 | -0.14 | -126.70 |
| H2.21/11.18 | 383.44 | 7.56 | 324.65 | 1.86 | 128.80 |
| H2.21/11.19 | 358.92 | 2.77 | 476.09 | 3.05 | 493.62 |
| H2.21/11.2 | 176.94 | 2.31 | 77.90 | 0.86 | 49.30 |
| H2.21/11.20 | 600.19 | -0.69 | 372.15 | 2.36 | 197.55 |
| H2.21/11.21 | 244.44 | 5.06 | 302.15 | 3.11 | 264.30 |
| H2.21/11.22 | 333.19 | 4.31 | 232.90 | 1.11 | 45.80 |
| H2.21/11.23 | 348.76 | 5.66 | 469.79 | 1.06 | 281.27 |
| H2.21/11.24 | -94.09 | -0.32 | -36.13 | 0.20 | -17.25 |
| H2.21/11.25 | 305.91 | 3.68 | 338.87 | 2.20 | 254.75 |
| H2.21/11.26 | 155.91 | 5.01 | 295.20 | 1.87 | 189.75 |
| H2.21/11.3 | 324.19 | 6.81 | 367.90 | 2.11 | 188.05 |
| H2.21/11.4 | 752.44 | 7.81 | 457.40 | 0.86 | 274.80 |
| H2.21/11.5 | 1235.94 | 9.31 | 1104.90 | 1.86 | 661.30 |
| H2.21/11.6 | 379.94 | 7.31 | 609.57 | 2.19 | 399.63 |
| H2.21/11.7 | 385.94 | 5.31 | 523.23 | 2.86 | 441.97 |
| H2.21/11.8 | 437.27 | 6.64 | 452.23 | 2.86 | 343.30 |
| H2.21/11.9 | 361.74 | 8.51 | 588.70 | 2.46 | 349.30 |
| H2.34/11 | 666.19 | 7.06 | 874.65 | 2.86 | 662.05 |
| H27.2/12 | 222.84 | 4.51 | 412.58 | 3.22 | 356.31 |
| H3.22/10 | 208.58 | 7.68 | 599.87 | 4.20 | 485.75 |
| H3.22/11 | 516.96 | 4.46 | 580.99 | 2.06 | 435.27 |
| H3.22/12 | 983.41 | 4.18 | 850.37 | 2.20 | 717.75 |
| H3.22/13 | 384.26 | 5.16 | 515.79 | 2.56 | 406.77 |
| H3.22/14 | 447.76 | 3.66 | 726.79 | 4.06 | 674.27 |
| H3.22/15 | 144.66 | 7.68 | 477.37 | 2.20 | 231.00 |
| H3.22/16 | 303.91 | 6.18 | 526.37 | 1.95 | 308.75 |
| H3.22/17 | 279.16 | 3.86 | 444.59 | 2.06 | 368.27 |
| H3.22/18 | 196.24 | 4.68 | 484.20 | 3.87 | 488.08 |
| H3.22/19 | 505.76 | 8.99 | 739.46 | 2.73 | 542.94 |
| H3.22/20 | 232.91 | 5.68 | 416.62 | 2.45 | 287.00 |
| H3.22/21 | 480.31 | 7.28 | 463.07 | 2.00 | 293.55 |
| H3.22/22 | 430.36 | 5.86 | 554.19 | 1.86 | 415.07 |
| H3.22/23 | 258.41 | 7.68 | 265.37 | 1.20 | 139.25 |
| H3.22/24 | 142.41 | 8.18 | 611.37 | 3.20 | 404.00 |
| H3.22/25 | 189.71 | 8.88 | 761.47 | 3.60 | 487.55 |
| H3.22/26 | 131.91 | 4.68 | 763.87 | 5.20 | 782.75 |
| H3.22/27 | 386.11 | 7.08 | 420.27 | 1.60 | 155.15 |
| H3.22/28 | 347.01 | 9.66 | 615.04 | 2.06 | 406.52 |
| H3.22/29 | 239.41 | 9.93 | 720.62 | 2.95 | 411.75 |
| H3.22/30 | 261.76 | 3.16 | 304.29 | 0.06 | 189.27 |
| H3.22/31 | 133.91 | 6.68 | 59.87 | 0.20 | -17.25 |
| H3.22/32 | 500.43 | 4.99 | 409.79 | 0.73 | 254.60 |
| H3.22/33 | 179.58 | 11.35 | 1025.20 | 4.20 | 691.75 |
| H3.22/34 | 631.76 | 6.66 | 744.79 | 3.56 | 614.77 |
| H3.22/35 | 296.76 | 4.66 | 509.29 | 1.06 | 311.27 |
| H3.22/36 | 140.58 | 8.35 | 755.54 | 2.87 | 389.08 |
| H3.22/37 | 314.01 | 3.41 | 357.29 | 0.81 | 236.02 |
| H3.22/38 | 31.91 | 1.68 | -4.13 | 0.20 | -17.25 |
| H3.22/39 | 493.43 | 5.33 | 450.46 | 1.39 | 296.60 |
| H3.22/40 | 41.78 | 0.60 | -20.16 | 0.33 | -35.24 |
| H3.22/6 | 263.91 | 8.01 | 503.87 | 2.87 | 285.08 |
| H3.22/7 | 431.76 | 5.16 | 547.79 | 2.56 | 406.77 |
| H3.22/8 | 10.91 | 4.68 | 374.87 | 3.20 | 344.75 |
| H3.22/9 | 242.91 | 5.68 | 708.87 | 5.20 | 705.75 |
| H4.2/12 | 1037.94 | 7.31 | 656.90 | 2.61 | 459.05 |
| H5.14/09.1 | 152.78 | 8.10 | 315.84 | 1.83 | 139.76 |
| H5.14/09.10 | 413.03 | 7.85 | 558.59 | 3.83 | 389.26 |
| H5.14/09.11 | 304.76 | 6.16 | 751.79 | 3.56 | 607.77 |
| H5.14/09.12 | 369.76 | 5.66 | 839.79 | 5.06 | 766.27 |
| H5.14/09.13 | 191.11 | 6.68 | 443.47 | 3.00 | 309.55 |
| H5.14/09.14 | 281.78 | 5.27 | 534.17 | 3.33 | 437.43 |
| H5.14/09.15 | -73.22 | 0.60 | -20.16 | 0.33 | -35.24 |
| H5.14/09.16 | 186.76 | 0.37 | 35.88 | -0.53 | -17.40 |
| H5.14/09.17 | 174.53 | 6.60 | 750.59 | 3.33 | 534.01 |
| H5.14/09.18 | 26.78 | 2.60 | 255.84 | 1.33 | 212.76 |
| H5.14/09.19 | 265.76 | 2.87 | 214.88 | 1.47 | 158.60 |
| H5.14/09.2 | 230.45 | 7.60 | 759.84 | 5.00 | 662.76 |
| H5.14/09.20 | 611.26 | 8.87 | 587.38 | 2.47 | 418.60 |
| H5.14/09.21 | 387.38 | 7.40 | 1031.84 | 4.53 | 798.56 |
| H5.14/09.22 | 274.76 | 4.37 | 79.88 | -0.53 | -17.40 |
| H5.14/09.23 | 703.09 | 0.37 | 35.88 | -0.53 | -17.40 |
| H5.14/09.24 | 484.09 | 8.04 | 223.21 | 0.80 | 60.60 |
| H5.14/09.25 | 690.09 | 3.04 | 359.21 | 0.47 | 258.60 |
| H5.14/09.26 | 543.76 | 6.70 | 815.88 | 4.14 | 675.60 |
| H5.14/09.27 | 265.76 | 6.37 | 479.88 | 1.47 | 317.60 |
| H5.14/09.28 | 548.76 | 2.87 | 38.88 | 0.47 | 141.60 |
| H5.14/09.29 | 715.26 | 9.37 | 872.88 | 3.97 | 672.60 |
| H5.14/09.3 | 746.76 | 0.66 | 256.79 | 0.06 | 189.27 |
| H5.14/09.30 | 115.76 | -0.34 | 241.79 | 0.06 | 189.27 |
| H5.14/09.4 | 479.26 | 6.16 | 673.79 | 3.06 | 547.27 |
| H5.14/09.5 | 400.01 | 12.91 | 773.54 | 2.81 | 480.02 |
| H5.14/09.6 | 507.78 | 13.60 | 459.84 | 1.33 | 156.76 |
| H5.14/09.7 | 692.76 | 8.66 | 1040.79 | 3.56 | 801.27 |
| H5.14/09.8 | 474.76 | 10.33 | 762.12 | 3.06 | 486.60 |
| H5.14/09.9 | 360.78 | 8.10 | 639.84 | 4.08 | 490.51 |
| H5.3/12.1 | -17.06 | -0.69 | -156.10 | -0.14 | -126.70 |
| H5.3/12.10 | 511.24 | 5.35 | 564.54 | 2.87 | 451.75 |
| H5.3/12.11 | 489.26 | 4.66 | 639.79 | 2.06 | 502.77 |
| H5.3/12.12 | 129.24 | 4.35 | 288.87 | 1.53 | 192.08 |
| H5.3/12.13 | 589.76 | 5.16 | 622.29 | 3.06 | 518.27 |
| H5.3/12.14 | 25.91 | 5.68 | 468.87 | 3.20 | 300.75 |
| H5.3/12.15 | 463.94 | 6.56 | 473.15 | 2.61 | 316.55 |
| H5.3/12.2 | 605.61 | 6.31 | 663.23 | 2.86 | 469.30 |
| H5.3/12.3 | 466.44 | 7.56 | 450.90 | 1.36 | 192.55 |
| H5.3/12.4 | 383.00 | 6.85 | 585.34 | 2.88 | 449.12 |
| H5.3/12.5 | 394.19 | 7.06 | 435.40 | 1.61 | 143.80 |
| H5.3/12.6 | 412.76 | 2.66 | 449.79 | 2.06 | 328.27 |
| H5.3/12.7 | 395.76 | 2.66 | 549.79 | 3.06 | 497.27 |
| H5.3/12.8 | 115.76 | -0.34 | 241.79 | 0.06 | 189.27 |
| H5.3/12.9 | 297.84 | 2.91 | 137.78 | 1.42 | 127.51 |
| H5.ej.10 | 927.07 | 11.48 | 1017.77 | 3.12 | 789.03 |
| H6.19/12 | 477.45 | 6.90 | 820.69 | 4.98 | 744.42 |
| H6.3/14 | 600.92 | 5.10 | 736.76 | 4.05 | 671.29 |
| H6.52.11 | 738.26 | 4.87 | 909.88 | 3.47 | 836.60 |
| H9.10.1 | 488.44 | 10.31 | 516.40 | 2.36 | 171.80 |
| H9.10.10 | 231.91 | 5.68 | 278.87 | 1.20 | 153.75 |
| H9.10.11 | 639.24 | 5.68 | 488.87 | 2.53 | 356.08 |
| H9.10.12 | 1199.91 | 7.18 | 1336.87 | 5.20 | 1266.25 |
| H9.10.2 | 445.26 | 3.66 | 417.79 | 1.56 | 291.27 |
| H9.10.3 | 385.44 | 6.56 | 172.40 | 0.86 | 14.30 |
| H9.10.4 | 228.91 | 4.18 | 465.37 | 3.20 | 456.75 |
| H9.10.5 | 625.94 | 7.64 | 653.90 | 3.19 | 445.97 |
| H9.10.6 | 337.56 | 4.46 | 457.79 | 1.86 | 328.67 |
| H9.10.7 | 105.91 | 2.68 | 10.87 | 0.20 | -17.25 |
| H9.10.9 | 493.94 | 8.31 | 551.23 | 3.19 | 302.97 |
| H9.13 | 231.17 | 7.24 | 423.78 | 3.09 | 288.84 |
| H9.32/11 | 139.91 | 4.01 | 272.87 | 2.20 | 247.42 |
| H9.48.11 | 871.58 | 10.43 | 1042.09 | 4.71 | 769.62 |
| H9.6/11 | 521.79 | 8.06 | 649.90 | 2.86 | 455.05 |
| H9.7.12 | 1125.61 | 7.98 | 587.23 | 2.53 | 363.97 |
| H9.9/12 | 222.76 | 5.66 | 527.29 | 2.06 | 392.27 |
| Indo1 | 592.34 | 8.66 | 878.53 | 5.17 | 749.76 |
| Indo10 | 685.84 | 7.58 | 753.45 | 3.42 | 575.18 |
| Indo11 | 344.25 | 6.85 | 947.09 | 4.13 | 810.37 |
| Indo12 | 348.17 | 4.24 | 430.45 | 2.09 | 359.84 |
| Indo13 | 408.59 | 6.41 | 336.53 | 1.67 | 214.01 |
| Indo14 | 395.59 | 3.91 | 691.78 | 3.42 | 664.76 |
| Indo15 | 767.84 | 7.91 | 514.78 | 2.42 | 252.51 |
| Indo16 | 276.25 | 5.10 | 401.09 | 3.05 | 374.29 |
| Indo17 | 549.58 | 6.43 | 454.42 | 2.71 | 313.62 |
| Indo18 | 254.84 | 2.91 | 156.78 | 1.42 | 115.51 |
| Indo19 | 515.51 | 7.58 | 1018.11 | 5.09 | 921.84 |
| Indo2 | 209.84 | 7.16 | 525.53 | 2.67 | 329.26 |
| Indo20 | 904.34 | 7.91 | 735.78 | 2.42 | 541.01 |
| Indo21 | 12.84 | -0.09 | 3.78 | 0.42 | 22.51 |
| Indo22 | 620.34 | 6.41 | 599.78 | 1.42 | 316.51 |
| Indo23 | 609.51 | 3.91 | 739.78 | 3.42 | 699.84 |
| Indo24 | 519.34 | 5.41 | 460.53 | 2.17 | 337.51 |
| Indo25 | 356.45 | 5.50 | 558.09 | 3.58 | 465.82 |
| Indo26 | 626.84 | 1.91 | 742.78 | 2.42 | 761.51 |
| Indo27 | 735.09 | 7.41 | 570.53 | 2.67 | 379.01 |
| Indo28 | 277.00 | 4.10 | 409.34 | 1.88 | 281.62 |
| Indo3 | 308.34 | 5.41 | 773.53 | 4.17 | 687.76 |
| Indo4 | -208.93 | -2.52 | -418.23 | -1.88 | -358.97 |
| Indo5 | 51.07 | 3.48 | 108.27 | 1.12 | 87.03 |
| Indo6 | 373.84 | 3.91 | 279.78 | 1.42 | 163.51 |
| Indo7 | 349.84 | 4.91 | 516.28 | 3.42 | 438.01 |
| Indo8 | 544.84 | 4.41 | 161.78 | 0.42 | 22.51 |
| Indo9 | 413.17 | 3.91 | 566.45 | 3.42 | 548.18 |
| JPN1 | 606.83 | 2.49 | 665.79 | 2.29 | -8.20 |
| JPN1.1144.2 | 532.28 | 9.10 | 954.84 | 3.33 | 733.26 |
| JPN10 | 537.03 | 13.29 | 708.59 | 3.49 | 412.80 |
| JPN11 | 284.53 | 5.29 | 716.09 | 1.99 | 530.80 |
| JPN12 | 294.78 | 10.29 | 764.59 | 2.99 | 510.80 |
| JPN13 | 917.03 | 5.29 | 449.59 | 1.49 | 351.80 |
| JPN14 | 391.92 | 9.43 | 888.42 | 4.05 | 629.29 |
| JPN15 | 979.03 | 3.62 | 810.59 | 2.49 | 751.47 |
| JPN16 | 627.40 | 2.15 | 448.10 | 1.79 | 445.36 |
| JPN17 | 636.07 | -0.52 | 90.10 | 0.12 | 149.36 |
| JPN18 | 240.57 | 1.98 | 65.77 | 0.12 | 53.53 |
| JPN19 | -208.93 | -2.52 | -418.23 | -1.88 | -358.97 |
| JPN2 | 121.00 | 9.35 | 849.09 | 4.63 | 689.62 |
| JPN20 | 339.07 | 10.81 | 483.44 | 2.12 | 184.03 |
| JPN21 | -208.93 | -2.52 | -418.23 | -1.88 | -358.97 |
| JPN22 | 269.32 | 8.48 | 289.77 | 1.62 | 138.78 |
| JPN23 | 1091.07 | 6.48 | 273.77 | 1.12 | 206.03 |
| JPN24 | 107.07 | 8.98 | -70.23 | -0.38 | -175.97 |
| JPN25 | 154.32 | 6.73 | 313.02 | 2.12 | 200.53 |
| JPN26 | 3.07 | 2.48 | 13.77 | 0.12 | -29.97 |
| JPN28 | 112.32 | 7.48 | 461.52 | 2.12 | 190.53 |
| JPN29 | 62.07 | 2.81 | -270.56 | -1.21 | -275.30 |
| JPN3 | 598.70 | 1.96 | 699.59 | 1.82 | 680.80 |
| JPN30 | 251.57 | 8.98 | 238.77 | 1.62 | 72.53 |
| JPN31 | -17.26 | 2.81 | -239.23 | -1.21 | -285.97 |
| JPN32 | 281.07 | 2.48 | 196.77 | 3.12 | 256.03 |
| JPN33 | 106.07 | 2.48 | -2.23 | 1.45 | -14.30 |
| JPN34 | -208.93 | -2.52 | -418.23 | -1.88 | -358.97 |
| JPN35 | 139.57 | 9.48 | 386.77 | 2.62 | 177.03 |
| JPN36 | 184.57 | 7.98 | 506.27 | 3.12 | 222.03 |
| JPN37 | 457.07 | 8.48 | 790.77 | 5.12 | 663.03 |
| JPN38 | 611.40 | 6.48 | 203.10 | 0.12 | -94.97 |
| JPN39 | 328.25 | 6.77 | 997.42 | 4.71 | 878.62 |
| JPN4 | -20.97 | -0.71 | 4.59 | -0.51 | -8.20 |
| JPN40 | 92.57 | 4.98 | 135.27 | 3.12 | 104.03 |
| JPN41 | 129.07 | 7.23 | 417.52 | 3.37 | 361.78 |
| JPN42 | 226.82 | 11.48 | 226.77 | 0.87 | -65.47 |
| JPN43 | 101.07 | 0.48 | 14.27 | -0.38 | -2.97 |
| JPN44 | 55.47 | 4.68 | 196.97 | 1.32 | 98.63 |
| JPN45 | 144.40 | 9.15 | -53.56 | -1.88 | -358.97 |
| JPN46 | -208.93 | -2.52 | -418.23 | -1.88 | -358.97 |
| JPN47 | 3.07 | 4.73 | 115.27 | 0.87 | -7.72 |
| JPN48 | 357.84 | 1.91 | 378.78 | 1.42 | 278.51 |
| JPN49 | 356.59 | 7.66 | 452.28 | 3.17 | 282.51 |
| JPN5 | 519.84 | 6.91 | 552.11 | 3.09 | 403.18 |
| JPN50 | 574.09 | 10.41 | 784.78 | 4.42 | 511.51 |
| JPN51 | 314.84 | 1.91 | 75.78 | 0.42 | 22.51 |
| JPN52 | 370.84 | 8.16 | 551.78 | 2.67 | 337.01 |
| JPN53 | 496.84 | 7.41 | 759.28 | 4.42 | 597.51 |
| JPN55 | 1075.34 | 6.91 | 453.28 | 3.42 | 386.51 |
| JPN56 | 26.07 | 1.48 | 90.77 | 0.79 | 105.03 |
| JPN6 | 308.03 | 5.79 | 1093.59 | 5.49 | 1063.30 |
| JPN7 | 1105.70 | 8.96 | 882.26 | 4.16 | 690.80 |
| JPN8 | 898.78 | 5.54 | 845.84 | 2.74 | 747.55 |
| JPN9 | 416.94 | 9.81 | 454.65 | 1.36 | 87.05 |
| Moz1 | 326.28 | 6.10 | 603.34 | 2.83 | 484.76 |
| Moz1.1 | -25.22 | -0.32 | -251.25 | -2.84 | -297.86 |
| Moz1.10 | -20.97 | -0.71 | 4.59 | -0.51 | -8.20 |
| Moz1.11 | 389.78 | 11.29 | 831.59 | 4.49 | 595.30 |
| Moz1.12 | 227.65 | 7.90 | 487.89 | 2.58 | 337.02 |
| Moz1.13 | 108.28 | 8.29 | 651.09 | 4.49 | 537.30 |
| Moz1.14 | 278.58 | 18.43 | 847.09 | 3.38 | 406.62 |
| Moz1.15 | 1361.03 | 5.79 | 1676.59 | 4.99 | 1642.30 |
| Moz1.16 | 124.03 | 5.96 | 228.92 | 0.16 | 51.47 |
| Moz1.17 | 221.45 | 7.10 | 569.89 | 3.38 | 448.22 |
| Moz1.18 | 668.03 | 4.29 | 763.59 | 1.49 | 601.80 |
| Moz1.19 | 410.92 | 13.10 | 948.09 | 5.71 | 677.95 |
| Moz1.2 | 186.76 | 0.37 | 35.88 | -0.53 | -17.40 |
| Moz1.20 | 35.53 | 4.79 | 77.59 | -0.51 | -8.20 |
| Moz1.21 | -20.97 | -0.71 | 4.59 | -0.51 | -8.20 |
| Moz1.22 | 287.70 | 7.96 | 481.59 | 1.82 | 221.13 |
| Moz1.23 | 485.78 | 13.43 | 621.50 | 3.66 | 260.14 |
| Moz1.24 | 337.50 | 10.60 | 787.09 | 4.38 | 551.62 |
| Moz1.25 | 820.53 | 3.29 | 509.59 | 1.99 | 431.80 |
| Moz1.26 | 339.75 | 11.10 | 1347.59 | 5.88 | 1018.12 |
| Moz1.27 | 290.03 | 14.29 | 1002.59 | 5.49 | 577.80 |
| Moz1.28 | -25.22 | -0.32 | -251.25 | -2.84 | -297.86 |
| Moz1.29 | 488.25 | 7.43 | 1037.09 | 4.38 | 859.29 |
| Moz1.3 | -25.22 | -0.32 | -251.25 | -2.84 | -297.86 |
| Moz1.30 | -20.97 | -0.71 | 4.59 | -0.51 | -8.20 |
| Moz1.31 | 426.36 | 6.62 | 780.59 | 4.16 | 649.13 |
| Moz1.32 | 275.53 | 7.79 | 789.59 | 5.49 | 1196.80 |
| Moz1.33 | -20.97 | -0.71 | 4.59 | -0.51 | -8.20 |
| Moz1.34 | 377.03 | 4.29 | 364.09 | 0.99 | 193.80 |
| Moz1.35 | 509.36 | 5.62 | 616.59 | 2.49 | 498.47 |
| Moz1.36 | 601.36 | 6.62 | 555.92 | 1.49 | 263.80 |
| Moz1.37 | 226.36 | 5.29 | 484.26 | 2.49 | 395.80 |
| Moz1.38 | 178.36 | 5.96 | 514.59 | 2.16 | 378.80 |
| Moz1.39 | 165.53 | 4.79 | 499.59 | 2.99 | 286.80 |
| Moz1.4 | 424.78 | 4.35 | 438.75 | 1.49 | 362.81 |
| Moz1.40 | 483.03 | 1.29 | 433.59 | 1.49 | 420.80 |
| Moz1.41 | -20.97 | -0.71 | 4.59 | -0.51 | -8.20 |
| Moz1.5 | 114.78 | 4.68 | 329.75 | 1.16 | 250.14 |
| Moz1.6 | -25.22 | -0.32 | -251.25 | -2.84 | -297.86 |
| Moz1.7 | 490.78 | 9.68 | 1087.75 | 2.16 | 942.14 |
| Moz1.8 | 439.82 | 13.73 | 692.02 | 2.12 | 216.78 |
| Moz1.9 | 1067.36 | 2.62 | 656.59 | 1.49 | 597.13 |
| Moz10 | 597.76 | 11.37 | 535.38 | 0.97 | 205.10 |
| Moz11 | 309.76 | 2.66 | 397.79 | 1.06 | 263.27 |
| Moz12 | 477.26 | 13.37 | 699.13 | 3.22 | 340.85 |
| Moz13 | 590.26 | 7.87 | 486.88 | 2.47 | 331.60 |
| Moz14 | 439.03 | 14.35 | 1364.34 | 4.33 | 877.01 |
| Moz15 | 188.45 | 7.93 | 399.51 | 2.00 | 146.76 |
| Moz16 | 284.45 | 9.60 | 1120.84 | 4.33 | 858.43 |
| Moz17 | 139.28 | 7.10 | 500.34 | 2.33 | 265.76 |
| Moz18 | 596.16 | 10.17 | 977.88 | 3.47 | 771.20 |
| Moz19 | 186.76 | 0.37 | 35.88 | -0.53 | -17.40 |
| Moz2 | 536.76 | 11.87 | 918.88 | 3.97 | 624.10 |
| Moz2.10 | 348.76 | 5.70 | 471.21 | 2.47 | 386.93 |
| Moz2.11 | 411.78 | 10.68 | 1144.75 | 4.16 | 917.14 |
| Moz2.12 | 784.78 | 14.68 | 188.75 | 1.16 | -2.86 |
| Moz2.13 | 711.78 | 7.68 | 440.75 | 1.16 | 314.14 |
| Moz2.14 | 304.45 | 19.35 | 59.08 | 0.16 | -29.53 |
| Moz2.15 | 529.78 | 3.01 | 192.75 | -0.84 | 117.47 |
| Moz2.16 | 356.78 | 7.60 | 1068.09 | 4.58 | 906.01 |
| Moz2.17 | 321.28 | 6.60 | 275.34 | 1.33 | 148.76 |
| Moz2.18 | 412.78 | 3.93 | 366.17 | 2.00 | 284.43 |
| Moz2.19 | 202.78 | 10.68 | 490.75 | 3.16 | 319.14 |
| Moz2.2 | 998.76 | 4.37 | 956.88 | 1.47 | 830.60 |
| Moz2.20 | 423.11 | 6.60 | 396.17 | 3.00 | 267.76 |
| Moz2.21 | 435.78 | 5.60 | 316.84 | 2.33 | 199.76 |
| Moz2.22 | 519.78 | 5.27 | 647.84 | 3.33 | 571.09 |
| Moz2.23 | 446.28 | 6.60 | 474.34 | 2.33 | 339.26 |
| Moz2.24 | 816.78 | 3.68 | 568.75 | 0.16 | 510.14 |
| Moz2.25 | 774.78 | 7.68 | 509.75 | 1.16 | 347.64 |
| Moz2.26 | -73.22 | 0.60 | -20.16 | 0.33 | -35.24 |
| Moz2.27 | 1694.78 | 3.68 | 986.75 | 1.16 | 940.14 |
| Moz2.28 | 610.78 | 6.68 | 196.75 | -0.84 | -58.86 |
| Moz2.29 | 43.78 | 5.68 | -134.25 | -2.84 | -297.86 |
| Moz2.3 | 446.03 | 10.85 | 991.59 | 5.08 | 748.01 |
| Moz2.30 | 315.78 | 15.60 | 1200.84 | 5.33 | 746.76 |
| Moz2.31 | 338.03 | 11.10 | 728.59 | 3.33 | 387.26 |
| Moz2.32 | 479.11 | 9.35 | 454.08 | 0.16 | 151.81 |
| Moz2.33 | 431.28 | 9.60 | 694.34 | 3.83 | 472.26 |
| Moz2.34 | 314.43 | 3.87 | 438.38 | 1.47 | 342.60 |
| Moz2.35 | 390.45 | 4.60 | 357.17 | 1.33 | 258.76 |
| Moz2.36 | 268.28 | 7.68 | 214.75 | 0.16 | 38.14 |
| Moz2.37 | 308.28 | 11.60 | 685.84 | 2.83 | 285.26 |
| Moz2.38 | 202.78 | 6.68 | -61.25 | -0.84 | -154.86 |
| Moz2.39 | 269.28 | 13.10 | 815.34 | 4.58 | 426.26 |
| Moz2.4 | 428.76 | 7.04 | 778.55 | 3.47 | 652.27 |
| Moz2.40 | 273.11 | 6.35 | 162.42 | -0.51 | 29.14 |
| Moz2.41 | 99.78 | 1.68 | -119.75 | -1.84 | -161.36 |
| Moz2.42 | 215.78 | 7.43 | -93.25 | -2.34 | -255.86 |
| Moz2.43 | 452.78 | 3.93 | 237.17 | 1.33 | 133.43 |
| Moz2.44 | 433.53 | 14.68 | 156.75 | -0.84 | -120.11 |
| Moz2.45 | 522.53 | 9.35 | 430.59 | 1.33 | 92.26 |
| Moz2.46 | 832.78 | 3.68 | 163.75 | -0.84 | 102.14 |
| Moz2.47 | 125.25 | 5.10 | 270.09 | 1.38 | 176.62 |
| Moz2.48 | 1072.78 | 5.68 | 217.75 | 1.16 | 94.14 |
| Moz2.49 | 232.28 | 10.68 | 358.75 | 1.66 | 127.14 |
| Moz2.5 | 115.76 | -0.34 | 241.79 | 0.06 | 189.27 |
| Moz2.50 | 529.45 | 8.35 | 150.75 | -0.84 | -94.86 |
| Moz2.51 | 501.00 | 8.10 | 457.59 | 2.63 | 305.12 |
| Moz2.52 | 269.58 | 8.48 | -9.85 | -2.04 | -212.66 |
| Moz2.53 | 348.27 | 8.64 | 255.57 | 1.86 | 105.97 |
| Moz2.54 | 238.58 | 6.28 | 212.35 | 1.36 | 100.74 |
| Moz2.55 | 396.44 | 8.81 | 436.40 | 3.86 | 267.30 |
| Moz2.56 | 154.25 | 7.10 | 303.09 | 1.38 | 176.62 |
| Moz2.57 | 255.00 | 5.35 | 475.59 | 3.13 | 425.62 |
| Moz2.58 | 1294.78 | 6.68 | 828.75 | 0.16 | 552.14 |
| Moz2.59 | -25.22 | -0.32 | -251.25 | -2.84 | -297.86 |
| Moz2.6 | 510.26 | 11.87 | 701.88 | 3.47 | 437.10 |
| Moz2.60 | -25.22 | -0.32 | -251.25 | -2.84 | -297.86 |
| Moz2.61 | -25.22 | -0.32 | -251.25 | -2.84 | -297.86 |
| Moz2.7 | 348.76 | 11.37 | 504.88 | 3.47 | 293.60 |
| Moz2.8 | 266.76 | 4.37 | 271.88 | 2.47 | 209.60 |
| Moz2.9 | 186.76 | 0.37 | 35.88 | -0.53 | -17.40 |
| Moz20 | 401.43 | 3.66 | 460.79 | 2.06 | 349.27 |
| Moz21 | 385.76 | 2.66 | 486.79 | 2.06 | 406.77 |
| Moz22 | 186.76 | 0.37 | 35.88 | -0.53 | -17.40 |
| Moz23 | 214.45 | 9.60 | 756.17 | 2.66 | 395.76 |
| Moz24 | 334.78 | 7.60 | 1013.84 | 4.66 | 817.43 |
| Moz25 | 1206.76 | 5.37 | 827.88 | 2.47 | 725.60 |
| Moz3 | 408.51 | 8.12 | 611.38 | 2.22 | 416.60 |
| Moz4 | 328.76 | 5.04 | 484.21 | 2.14 | 354.60 |
| Moz5 | 329.43 | 5.04 | 399.88 | 1.47 | 274.60 |
| Moz6 | 406.76 | 11.04 | 589.55 | 2.80 | 296.93 |
| Moz7 | 372.01 | 6.12 | 609.38 | 2.97 | 393.35 |
| Moz8 | 186.76 | 0.37 | 35.88 | -0.53 | -17.40 |
| Moz9 | 186.76 | 0.37 | 35.88 | -0.53 | -17.40 |
| SPM103 | 125.32 | 8.98 | 462.52 | 4.12 | 323.53 |
| SPO104 | 1081.03 | 2.79 | 660.09 | 2.99 | 897.30 |
| Significance | ** | ** | ** | * | ** |

^CK^ check variety, **significant at p<0.01, *significant at p<0.05, ^FW^ average foliage fresh weight per plant (g), ^RN^ average storage root number per plant, ^RW^ average storage root weight per plant (g), ^MRN^ marketable storage root number per plant and ^MRW^ marketable storage root weight per plant (g)

Supplementary Table S6. Average values for yield and yield related traits in the study of 75 sweet potato genotypes (including four check varieties) during the second season in Bogura, Bangladesh

| Genotype ID | Genotypes | FW | RN | RW | MRN | MRW | RL | RB | DM |
| --- | --- | --- | --- | --- | --- | --- | --- | --- | --- |
| H1 | Moz1.32 | 624 | 6.8 | 417 | 6.1 | 360 | 13.6 | 15.9 | 22.8 |
| H2 | Moz1.7 | 590 | 3.73 | 325 | 4.47 | 325 | 11.7 | 15.4 | 22.6 |
| H3 | H6.52.11 | 986 | 2.47 | 126 | 1.4 | 102 | 12.2 | 17.1 | 25 |
| H4 | JPN6 | 788 | 5.07 | 242 | 3.4 | 213 | 12.3 | 17.1 | 23.2 |
| H5 | Moz1.26 | 756 | 4.17 | 265 | 2.67 | 234 | 14.5 | 15.6 | 23.4 |
| H6 | H9.7.12 | 654 | 6.6 | 310 | 4.53 | 274 | 10 | 14.2 | 23.3 |
| H7 | Moz2.27 | 856 | 4 | 401 | 3 | 401 | 14.4 | 17.2 | 24.4 |
| H8 | H5.ej.10 | 616 | 3.9 | 267 | 2.37 | 249 | 14.5 | 16.7 | 23.1 |
| H9 | H9.10.12 | 616 | 5.9 | 488 | 4.3 | 457 | 11.8 | 15.8 | 22.9 |
| H10 | Moz2.11 | 528 | 1.2 | 132 | 1.2 | 132 | 14 | 15 | 23.8 |
| H11 | Moz2.16 | 862 | 5.8 | 360 | 3.63 | 307 | 12.5 | 18.6 | 22.7 |
| H12 | JPN39 | 706 | 3.87 | 381 | 2.53 | 256 | 13.6 | 16.9 | 22.8 |
| H13 | Moz14 | 756 | 6.47 | 389 | 4.43 | 370 | 12.4 | 17.7 | 23.2 |
| H14 | Moz1.29 | 668 | 1.5 | 29.5 | 0.1 | 3.4 | 6.7 | 14.9 | 24 |
| H15 | Moz16 | 414 | 1.5 | 25.2 | 0.1 | 5.6 | 9.1 | 12 | 25.8 |
| H16 | H5.3/12.7 | 586 | 4.67 | 391 | 3.33 | 366 | 12.9 | 16.8 | 22.2 |
| H17 | Moz2.2 | 528 | 3.07 | 230 | 2.33 | 218 | 13.3 | 16 | 22.4 |
| H18 | Moz24 | 650 | 6.17 | 467 | 4.8 | 435 | 12.7 | 17.4 | 22.3 |
| H19 | Indo11 | 138.6 | 4.47 | 355 | 2.7 | 310 | 12.7 | 16.6 | 23.9 |
| H20 | Moz1.15 | 748 | 5.97 | 260 | 4.73 | 252 | 12.5 | 15.8 | 24.7 |
| H21 | H5.14/09.21 | 700 | 5.37 | 325 | 3.97 | 298 | 13.4 | 17.8 | 24.4 |
| H22 | H3.22/26 | 640 | 6.03 | 330 | 4.2 | 301 | 12.5 | 16.8 | 25.2 |
| H23 | Moz18 | 736 | 4.53 | 236 | 2.63 | 206 | 11.7 | 16.6 | 25.1 |
| H24 | H5.14/09.12 | 504 | 1.9 | 231 | 1.8 | 264 | 12 | 17.1 | 23.2 |
| H25 | Indo26 | 652 | 6.4 | 381 | 3.73 | 341 | 13.3 | 16.5 | 25.1 |
| H26 | JPN15 | 960 | 5.63 | 432 | 4.07 | 378 | 14.4 | 15.7 | 27.6 |
| H27 | Indo1 | 472 | 4.77 | 236 | 3.27 | 198 | 10 | 18.2 | 24.6 |
| H28 | Moz2.3 | 514 | 3.53 | 217 | 2.73 | 211 | 8.27 | 14.6 | 23 |
| H29 | JPN8 | 714 | 6.1 | 420 | 4.77 | 389 | 11.4 | 17 | 22 |
| H30 | Moz2.30 | 634 | 6.9 | 504 | 5.13 | 468 | 13.1 | 16.6 | 22.6 |
| H31 | H6.19/12 | 438 | 4.27 | 398 | 3.17 | 388 | 10.6 | 15.9 | 22.5 |
| H32 | JPN1.1144.2 | 636 | 7.93 | 545 | 6.63 | 518 | 14.4 | 17.9 | 22.8 |
| H33 | Moz25 | 758 | 6.47 | 404 | 1.1 | 152 | 14.7 | 16.2 | 24.1 |
| H34 | H3.22/12 | 668 | 7.33 | 440 | 5.67 | 412 | 14.1 | 15.9 | 22.2 |
| H35 | BARIMistialu-15^CK^ | 706 | 5.73 | 518 | 4.17 | 477 | 14.1 | 17.2 | 22.2 |
| H36 | H3.22/9 | 582 | 4.73 | 351 | 4 | 338 | 11.1 | 16.7 | 23.2 |
| H37 | BARI_Mistialu-12^CK^ | 830 | 6 | 447 | 4.6 | 390 | 12.2 | 12.8 | 21.6 |
| H38 | H3.22/33 | 728 | 5.9 | 353 | 4.83 | 325 | 11.5 | 15.9 | 22.9 |
| H39 | BARIMistialu-14^CK^ | 696 | 5.77 | 408 | 9.83 | 584 | 14.3 | 15.9 | 22.2 |
| H40 | Indo19 | 576 | 6.33 | 383 | 5.43 | 397 | 13.1 | 13.3 | 23.1 |
| H41 | Moz1.9 | 700 | 7.2 | 456 | 16.7 | 404 | 15 | 15.2 | 22.2 |
| H42 | BARIMistialu-8^CK^ | 618 | 4 | 262 | 1.8 | 259 | 14.1 | 15.6 | 22.9 |
| H43 | H9.48.11 | 594 | 5.23 | 372 | 4.1 | 349 | 13.8 | 16.2 | 22.3 |
| H44 | Indo3 | 646 | 5.9 | 490 | 4.73 | 468 | 13.4 | 13.6 | 21.2 |
| H45 | H5.14/09.7 | 548 | 4.67 | 397 | 4.1 | 384 | 11.8 | 17.8 | 22.3 |
| H46 | JPN3 | 536 | 5.2 | 406 | 4.13 | 395 | 10.2 | 19.5 | 23.1 |
| H47 | Indo23 | 556 | 5.23 | 472 | 4.23 | 455 | 11.6 | 18.9 | 22.6 |
| H48 | Moz1.19 | 638 | 6.23 | 426 | 5.17 | 414 | 12.4 | 17.2 | 22.9 |
| H49 | H16.ej.10 | 858 | 5.57 | 547 | 4.17 | 499 | 12.1 | 17.2 | 22.9 |
| H50 | JPN7 | 658 | 4.6 | 430 | 4.9 | 356 | 10.7 | 17.6 | 22.6 |
| H51 | H5.14/09.26 | 622 | 7.03 | 481 | 5.43 | 423 | 10.4 | 17.3 | 21.7 |
| H52 | JPN2 | 554 | 4.43 | 324 | 3.3 | 288 | 7.63 | 15.7 | 23.2 |
| H53 | H3.22/14 | 530 | 7.63 | 371 | 5.23 | 353 | 13.8 | 15.8 | 24.1 |
| H54 | H5.14/09.29 | 642 | 7.47 | 399 | 4.3 | 325 | 13.9 | 17.6 | 26.1 |
| H55 | H6.3/14 | 512 | 5.4 | 372 | 4 | 336 | 11.3 | 17.5 | 24.4 |
| H56 | Indo14 | 602 | 5.5 | 383 | 3.7 | 323 | 15.3 | 17.2 | 24.5 |
| H57 | JPN37 | 560 | 6.17 | 379 | 5.06 | 375 | 12.3 | 17.7 | 24 |
| H58 | H5.14/09.2 | 506 | 5.47 | 351 | 4.7 | 336 | 12.9 | 18.3 | 23.9 |
| H59 | H2.34/11 | 608 | 5.63 | 563 | 4.87 | 555 | 12.6 | 18.6 | 25 |
| H60 | H2.21/11.5 | 664 | 2.53 | 153 | 1 | 62.7 | 13.6 | 16.2 | 26.3 |
| H61 | SPO104 | 628 | 5.67 | 404 | 4.47 | 382 | 12.8 | 16.6 | 22.6 |
| H62 | Moz1.31 | 614 | 5.6 | 344 | 4.23 | 288 | 11.6 | 15.7 | 22.3 |
| H63 | SPM103 | 618 | 6.07 | 436 | 4.47 | 398 | 13.7 | 17.9 | 22.2 |
| H64 | JPN14 | 540 | 5.53 | 320 | 3.43 | 248 | 10.9 | 16.1 | 23.3 |
| H65 | Moz2.4 | 500 | 5.63 | 347 | 3.63 | 311 | 10.6 | 17.6 | 23.5 |
| H66 | H3.22/34 | 676 | 6.53 | 459 | 4.57 | 416 | 11.5 | 19 | 23.3 |
| H67 | H5.14/09.11 | 652 | 5.8 | 473 | 3.93 | 411 | 12 | 16.4 | 22.8 |
| H68 | Moz1.18 | 440 | 4.67 | 352 | 3.53 | 409 | 12.6 | 19 | 22.2 |
| H69 | JPN53 | 492 | 6.53 | 431 | 4.67 | 411 | 11.2 | 16.1 | 22.3 |
| H70 | Moz1.11 | 590 | 6.5 | 341 | 5.5 | 279 | 11.7 | 15.1 | 23.2 |
| H71 | Moz1.27 | 922 | 6.1 | 400 | 4.2 | 397 | 12.6 | 17.3 | 21.5 |
| H72 | Indo10 | 916 | 5.6 | 364 | 4.3 | 315 | 13.4 | 17.4 | 24.7 |
| H73 | Moz2.22 | 648 | 5.87 | 497 | 4.8 | 467 | 10.8 | 18.9 | 22.6 |
| H74 | Moz2 | 552 | 6.5 | 571 | 5.43 | 546 | 11.5 | 16.6 | 23.3 |
| H75 | Moz2.58 | 748 | 6.83 | 334 | 3.73 | 266 | 12.3 | 17.1 | 32.6 |
| Significance | | * | *** | *** | * | *** | ** | NS | *** |

^CK^ check variety, ***significant at p<0.001, **significant at p<0.01, *significant at p<0.05, NS= non-significant, ^FW^ average foliage fresh weight per plant (g), ^RN^ average storage root number per plant, ^RW^ average storage root weight per plant (g), ^MRN^ marketable storage root number per plant, ^MRW^ marketable storage root weight per plant (g), ^RL^ average storage root length (cm), ^RD^ average storage root diameter (cm) and ^DW^ dry weight of storage roots (%)

Supplementary Table S7. Average values of 11 sweet potato genotypes across three environments for yield and quality traits during the final year

| Marketable storage root yield (MRY) (t/ha) | | | |
| --- | --- | --- | --- |
| Genotypes | Environments | | |
|  | ENV1 | ENV2 | ENV3 |
| H20 | 39.84abc | 56.33a | 58.04a |
| H3 | 39.34a-d | 55.76a | 47.11b |
| H37^CK^ | 39.35a-d | 46.48bc | 48.04b |
| H41 | 36.44bcd | 48.39b | 58.04a |
| H43 | 43.09a | 47.50bc | 47.59b |
| H49 | 35.82cde | 43.70c | 42.37c |
| H6 | 32.14ef | 37.52d | 35.37d |
| H61 | 35.58de | 47.54bc | 57.22a |
| H63 | 30.92f | 36.94d | 35.26d |
| H8 | 40.18ab | 44.65bc | 44.93bc |
| H9 | 39.74abc | 47.91b | 47.22b |
| Dry weight of storage roots (DW) (%) | | | |
| H20 | 23.05c | 23.85d | 22.99cd |
| H3 | 23.12c | 22.56d | 23.67cd |
| H37^CK^ | 35.99a | 36.22a | 29.71ab |
| H41 | 32.89a | 34.18ab | 30.33a |
| H43 | 22.40c | 22.66d | 22.96cd |
| H49 | 28.09b | 28.96c | 25.63bc |
| H6 | 31.96ab | 31.85bc | 30.29a |
| H61 | 19.31c | 22.00d | 19.54d |
| H63 | 21.16c | 22.74d | 21.84cd |
| H8 | 23.21c | 23.47d | 24.58c |
| H9 | 19.78c | 20.00d | 23.56cd |
| Beta-carotene content (BC) (mg/100g) | | | |
| H20 | 8.40de | 9.35de | 8.00d |
| H3 | 12.94cd | 13.65cd | 11.05cd |
| H37^CK^ | 16.94bc | 17.83bc | 12.48bcd |
| H41 | 8.39de | 9.54de | 13.04bcd |
| H43 | 6.68e | 6.65e | 12.52bcd |
| H49 | 9.44de | 10.58de | 15.50bc |
| H6 | 13.76cd | 13.99cd | 16.41bc |
| H61 | 12.97cd | 12.33cd | 14.13bc |
| H63 | 9.00de | 11.83de | 13.60bc |
| H8 | 49.12a | 48.76a | 38.86a |
| H9 | 20.77b | 22.25b | 16.95a |
| Ascorbic acid (vitamin C) content (VC) (mg/100g) | | | |
| H20 | 5.48c | 5.23c | 4.18b |
| H3 | 5.26c | 5.56c | 4.57b |
| H37^CK^ | 4.28c | 4.81c | 5.23b |
| H41 | 23.28a | 24.51a | 14.46b |
| H43 | 5.12c | 5.67c | 5.35b |
| H49 | 3.98c | 5.02c | 4.61b |
| H6 | 8.76bc | 9.56bc | 4.42b |
| H61 | 11.18b | 11.53b | 14.69a |
| H63 | 4.92c | 5.16c | 7.30b |
| H8 | 4.52c | 5.32c | 7.04b |
| H9 | 4.86c | 5.28c | 4.11b |

^CK^ check variety, values in columns with different letter(s) are significantly different at *p ≤* 0.05
